# Motor noise is rich signal in autism research and pharmacological treatments

**DOI:** 10.1038/srep37422

**Published:** 2016-11-21

**Authors:** E. B. Torres, K. Denisova

**Affiliations:** 1 Department of Psychology, Rutgers University, Department of Computer Science, Rutgers University, and Rutgers University Center for Cognitive Science, New Brunswick, NJ 08854, USA; 2 Department of Psychiatry, Columbia University College of Physicians and Surgeons, Sackler Institute for Developmental Psychobiology, Columbia University, New York, NY, 10032, USA; 3 Division of Developmental Neuroscience, New York State Psychiatric Institute, New York, NY, 10032, USA

## Abstract

The human body is in constant motion, from every breath that we take, to every visibly purposeful action that we perform. Remaining completely still on command is a major achievement as involuntary fluctuations in our motions are difficult to keep under control. Here we examine the noise-to-signal ratio of micro-movements present in time-series of head motions extracted from resting-state functional magnetic resonance imaging scans in 1048 participants. These included individuals with autism spectrum disorders (ASD) and healthy-controls in shared data from the Autism Brain Imaging Data Exchange (ABIDE) and the Attention-Deficit Hyperactivity Disorder (ADHD-200) databases. We find excess noise and randomness in the ASD cases, suggesting an uncertain motor-feedback signal. A power-law emerged describing an orderly relation between the dispersion and shape of the probability distribution functions best describing the stochastic properties under consideration with respect to intelligence quotient (IQ-scores). In ASD, deleterious patterns of noise are consistently exacerbated with the presence of secondary (comorbid) neuropsychiatric diagnoses, lower verbal and performance intelligence, and autism severity. Importantly, such patterns in ASD are present whether or not the participant takes psychotropic medication. These data unambiguously establish specific noise-to-signal levels of head micro-movements as a biologically informed core feature of ASD.

Humans are naturally variable in thought, behaviour and action across the spectrum of health and illness. However, individual variability cannot be examined precisely using conventional statistical approaches that de-emphasize individual differences by, for example, assuming normality and homogeneity of the data—a stumbling block for progress in neurodevelopmental research including phenotypically and genetically heterogeneous Autism Spectrum Disorders (ASD). Implementing the recent initiative of Precision Medicine^1^, for example, would require a conceptually novel, individualized statistical framework that would facilitate linkage between different layers of information across the knowledge network (Fig. 1A). Here we focus on characterizing the spontaneous physiological signals that underlie all individuals’ involuntary movements using an approach that harnesses the heretofore wayward individual variability in order to discover core biological signatures of the human nervous system indicative of its state of “health” or “illness”.

In typically developing individuals, a certain degree of variation exists in natural movements across multiple levels of conscious and unconscious awareness and control (Fig. 1B)^2^. Minute fluctuations in motor performance inevitably occur across different contexts, whether we intentionally move or whether the movements take place spontaneously and largely beneath awareness (Fig. 1C). Excess or deficits in involuntary motor variations relative to normative scales is undesirable, and has been found in the context of goal directed reaches^3^, decision making^4^ and gait patterns^5^ across various clinical populations with pathologies of the nervous system, including ASD^3^,^6^,^7^,^8^,^9^,^10^. Importantly, subtle fluctuations in the movement signal generate and carry new signals in a returning afferent stream: a form of re-entrant sensory feedback from the PNS to the CNS^11^, putatively conveying sensory feedback linked to self-generated movements. Consistent with theories of internal models for action (IMA)^12^, this form of (peripheral) returning signal would inform the CNS of the moment-by-moment accumulation of sensory evidence to help predict with a degree of certainty the sensory consequences of impending decisions and actions^11^. Without a reliable and predictable movement-returning signal across a variety of actions (Fig. 1C), the balance facilitating the continuous volitional control of one’s actions may be disrupted. Random accumulation of noisy signals would indicate difficulty in appropriately applying previously experienced information, thus making uncertain or obfuscating the sensory consequences of the impending actions in individuals with ASD.

The subtle nature of these fluctuations confers analytical difficulty when aiming to characterize potential variations in the signal, especially with regard to the study of populations with atypical neurodevelopment. For example, conventional analytical methods may arbitrarily predefine epochs in the signals and average the fluctuations in performance under *a priori* assumptions of normality. This is a concern as this approach results in smoothing out as noise potentially important (e.g., variability-related) components of the physiologically-related waveforms possibly reflecting underlying properties of the nervous systems of individuals with ASD^6^.

Albeit non-invasive brain imaging techniques, including resting-state functional magnetic resonance imaging (rs-fMRI), have the potential to reveal the brain-basis of neuropsychiatric and neurodevelopmental disorders, it is necessary to require the patient (and the control participants) to curtail overt behaviour and be motionless at some stage of the study. For instance, Electroencephalographic (EEG) data acquisition and inference is susceptible to artefacts due to participants’ eye blinks, and fMRI experiments require maximal damping of head movements that may occur during the scanning session while lying inside the scanner so as to prevent artefacts that emerge due to spontaneous movements^13^,^14^,^15^. Even upon padding the head during the scan in order to minimize movement, these minute fluctuations are detectable and known to confound the data if no cancellation procedures are in place^13^,^16^,^17^,^18^, often necessitating removal of entire datasets from statistical analysis.

Of particular relevance to the issue of movement-induced artefacts have been the recent works by Power and colleagues and Tyska and collegues pointing out the potential confounding effects of head motion on brain connectivity analyses in general^14^, but in particular those related to ASD^15^. Furthermore, recent work addressing general problems with some fMRI studies owing to the “*black box*” treatment of various stages of data processing point out the importance of not making a priori statistical assumptions about the underlying stochastic features of the data^19^. In the present study we follow up on these two general contemporary issues raised by these research groups in relation to data processing, statistical inference and interpretation. The first component refers to motor signatures extracted from head motions during rs-fMRI sessions. Specifically, we harness information from neuroimaging volumes that would normally be discarded. We do so in order to derive a new motor signature descriptor of involuntary motions of rs-fMRI data. The second component refers to the use of a new statistical platform for the personalized study of nervous systems disorders in the context of Precision Medicine^3^. This new platform makes no *a priori* assumption about the population statistic but rather addresses a posteriori what most likely the stochastic properties of the data may be.

Our approach stems from the motivation to connect multiple layers of information within the context of the knowledge network of the Precision Medicine paradigm (Fig. 1), including clinical records and behavioral descriptors that have yet to be objectively characterized.

The advent of publicly available databases containing original, motion-uncorrected neuroimaging (rs-fMRI), clinical and demographic data, combined with the recent work introducing a new data type and analytical techniques that examine the noise-to-signal ratio (NSR) signatures of bodily and neural rhythms^3^,^6^, may facilitate the objective characterization of involuntary motor signatures in large cross-sections of the population. Specifically, this work uses image-based estimation of head movements during the scan according to motion estimation methods validated relative to real physical movement, on the order of 100 μm^20^,^21^.

The head movement data obtainable from a large number of (rs-fMRI) datasets in the Autism Brain Imaging Data Exchange (ABIDE) database^22^ and in the Attention Deficit Hyperactivity Disorder (ADHD-200) database (altogether containing datasets of over 1500 individuals with and without ASD) is used here to characterize normative head movement data and better profile ASD and associated comorbid conditions. We investigate if fundamental differences manifest across different layers of this somatic motor signal across different kinematic parameters possibly separating ASD from typically developing controls.

For the purpose of our new analyses we first obtain head movement estimates using all original volumes during the scan (e.g., without excising certain volumes with excessive motion spikes). These time series of discrete movement samples (and not the 3D volume image data itself) are then transformed into time series of speed signals. A waveform derived from these time series is used to represent a continuous random process under the general rubric of Poison Random Process (PRP). To be more precise, we treat the spikes in the first rate of change in head motion as spikes of random amplitudes and random times. To model them, we build on previous research^6^ whereby the amplitudes and inter-spike interval times are modeled as independent and identically distributed (iid) random variables following a Gamma distribution. This framework has proven amenable to computational tractability, facilitating both inference^4^,^6^,^23^,^24^ and interpretation of the results. As such, the Gamma process is used here in combination with a waveform (coined “micro-movements”) representing the fluctuations in amplitude and timing of the spike trains derived from the rate of change in head positions and orientations. Further the spike trains thus defined are properly standardized to account for allometric effects of anatomical disparities across different ages. This normalization step is necessary to examine the motion data in relation to the clinical scores from cross-sections of the population comprised of individuals at different developmental stages. Within this general statistical framework, we ask if the somatic-motor disruptions that have been systematically quantified in ASD during voluntary behaviours across the body^6^ are also present in the involuntary fluctuations of participants’ head movements.

*Instead of predefining the hypothesis to test*, *as it is traditionally done*, *under the new approach one would let the inherent stochastic properties of the data automatically reveal the population trends*.

*What could these involuntary movements—considered a nuisance for statistical inference today—reveal about ASD?*

We establish the presence of atypical NSR in the motor signatures of individuals with ASD, including those individuals who were ‘off’ medication at the time of the scan. We further link the statistical features of the head motions to important individual-level features, with the atypically higher noise levels present in ASD individuals across different age groups, multiple levels of clinical severity, in the presence or absence of comorbidities, and across levels of IQ. Our findings thereby reveal a biologically informed core feature of atypical neurodevelopment in humans.

## Results

### Noise and randomness of involuntary micro-movements in ASD

**Figure 2:**
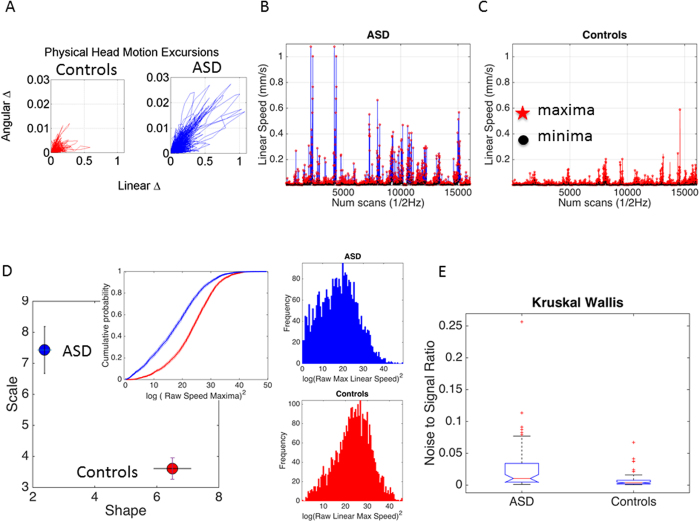
Excess in incremental head motion excursions (linear and angular) in ASD participants relative to typically developing controls during rs-fMRI session, with sample statistical methods to characterize their micro-movements’ NSR. (**A**) Excursions of increments in head displacements and head rotations plotted across all CT and ASD participants in UM_1 study-site obtained by taking the frame-by-frame difference along each position and orientation parameter and plotting the corresponding incremental pairs. (**B**) The magnitude of the rate of change of linear head displacement over time (speed scalar profile) is plotted in panels B and C for the 110 participants pooling the motion data over the dataset from one site (UM_1, 300 frames per participant) taken every 2 seconds. The landmarks of interest in this time-series are the speed maxima and minima. Panels (B,C) show time-series for ASD (N = 55) and CT (N = 55) participants, respectively. (**D**) The frequency histograms of the squared log of the raw maxima speeds are presented for each group along with the corresponding empirical cumulative probability distribution plots (note tight 95% Confidence Intervals (CIs)). The corresponding estimated parameters of the continuous Gamma family of probability distributions, the estimated shape and the estimated scale (NSR) from the empirical data, are plotted for each group on the Gamma plane (95% CIs). Note the unambiguous differences in stochastic signatures between the two groups. (**E**) The NSR estimated from the squared log of the raw max linear speed is significantly different according to the Kruskal-Wallis (non-parametric one-way ANOVA) test at the 0.01 alpha level (see main text for details).

Figure 2A illustrates the magnitudes of linear and angular incremental head displacements also shown in Supplementary Figures S1, S3. Figure 2B shows the rates of change of linear displacements, shown here for UM_1 site, using pooled data across all participants within each of the ASD and control (CT) groups. First, panels B and C in Fig. 2 show qualitative differences in the magnitude of the raw, scan-by-scan head motions between ASD and CT participants, whereby ASD participants have noticeably higher and more frequent fluctuations in speed peak amplitude per unit time. We quantified the differences in the raw speed peaks. In particular we refer the reader to the larger number of peaks with lower values in ASD (to the left of the graph) and the corresponding differences in the slope of the cumulative frequency histograms (inset in 2D). These cumulative frequency histograms obtained under similar sampling resolution and time duration of the section show higher accumulation of the peaks per unit time in the ASD group. Note that the squared log of the raw peak speed values was used for better visualization of the significant statistical disparity seen in Panel A. The differences between the empirical cumulative distribution functions (eCDFs) for these two empirical samples were statistically significant (Kolmogorov-Smirnov, test P < 10^−17^). The empirically estimated Gamma shape and scale parameters are also shown in Fig. 2D (plotted on the Gamma plane with 95% Confidence Intervals, CIs). This unambiguous quantitative difference between ASD and controls in the NSR was captured using non-parametric one-way ANOVA (the Kruskall-Wallis test) yielding statistically significant differences (*df* column, error, total (1, 108, 109), Γ^2^ 17.2, P > Γ^2^ 3.38 × 10^−5^).

**Figure 3:**
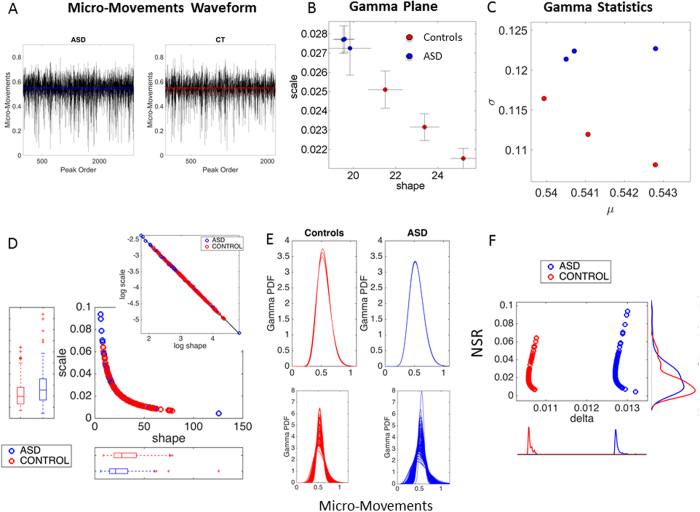
Micro-movements waveform analyses (extracted from LS spike trains) using data across 3 different sites (UM_1, UM_2, USM) of ABIDE. (**A**) Sample (normalized) micro-movements waveform from one site (USM) representing fluctuations in LS spike amplitude obtained from the 3D-linear displacements of the head during one scanning session. Lines plot the median values (ASD 0.5514, CT 0.5501; the Gamma estimated mean values ASD 0.5407, CT 0.5411 relative to dashed line 0.5). (**B**) The empirically estimated shape and scale Gamma parameters of the micro-movements waveform in (**A**) plotted on the Gamma parameter plane for each study-site. Note the distinct locations on the plane for ASD and CT groups from each study-site (95% Confidence Intervals). (**C**) Estimated Gamma mean and variance. (**D**) Individual participants’ data: all 246 participants (126_ASD_,120_CT_). Log-log plot of the shape, scale plane values reveal a power-law relation in the data (see details in the main text). Despite overlapping regions, the box plots reveal significant differences in both estimated Gamma parameters between the two groups. (**E**) Estimated PDFs of the grouped data (top) and obtained across all participants (bottom) in each of the two groups (ASD, CT) from all 3 study-sites. (**F**) Scatter plots and histograms of the NSR as a function of the fitting residual (denoted delta) from the power fit in (**D**) separate the two group types and hints at an ASD subset with much higher noise levels than controls.

Gamma parameter estimation on micro-movements waveform revealed statistically significant differences between groups in the NSR and in the shape values within each dataset that we analyzed. Figure 3A shows sample waveforms of the micro-movements (linear speed) for the USM site. (B) Panel shows the estimated stochastic signatures for each of the three main sites (see Methods section for explanation of steps and Supplementary Figures S3–S5). Across the 3 main studies (UM_1, UM_2 and USM) it was possible to differentiate ASD from CT participants as the Gamma parameters and the estimated Gamma mean and variance separated these groups (shown in panel 3C). Note that these group differences held independently for NYU, UCLA_1, OLIN, and PITT sites and are presented for both linear speed (LS) and angular speed (AS) micro-movements (Supplementary Figure S6).

Figure 3D presents individualized empirically estimated signatures for all participants from the 3 main studies of comparable temporal resolution and scan duration. Using this large group (N = 246; includes 126 ASD and 120 CT participants) we found a power law relation *f(x*) = *a* ·*x*^*b*^ between the log-log scale (*a*) and shape (*b*) estimated Gamma parameters with corresponding 95% confidence intervals (*a* = 0.53 [0.52, 0.54], b = −0.99 [−1.0, −0.98], goodness of fit SSE 1.49e-05 and adjusted R^2^ 0.99, RMSE 0.00037). Statistically significant differences for both estimated parameters were confirmed between ASD and CT (Friedman test, *df* columns, interaction, error, total (1, 1, 216, 219), Γ^2^ 163.51, P > Γ^2^ 1.93 × 10^−37^). The results of the NSR comparison using scatter and box plots are shown in Fig. 3D. Figure 3E shows the estimated probability density functions for each participant in the two groups. This figure underscores the differences between the variability patterns of LS micro-movements between ASD and CT participants.

The normal distance from each point representing a participant’s stochastic signature to the unit line from the power law relation characterizing the scatter on the Gamma plane was obtained (denoted delta) and the NSR plotted as a function of this residual value in Fig. 3F. Table 1 in the supplementary material lists the *p-values* of the ranksum Wilcoxon test comparing the median of the delta residual for ASD and CT taken for each study included in Fig. 3 (all P < 0.001). Supplementary Figure S7 reports the results for shape (p < 0.002) and scale (p < 0.001).

### Noisy cluster within ASD group

The analyses of the NSR revealed in the ASD group a subset of individuals with higher noise levels than that in the CT group (i.e., with NSR above 0.06, about 2.5 standard deviations from the estimated mean, in Fig. 3F). Closer inspection of this ratio revealed higher levels of variability in ASD.

**Figure 4:**
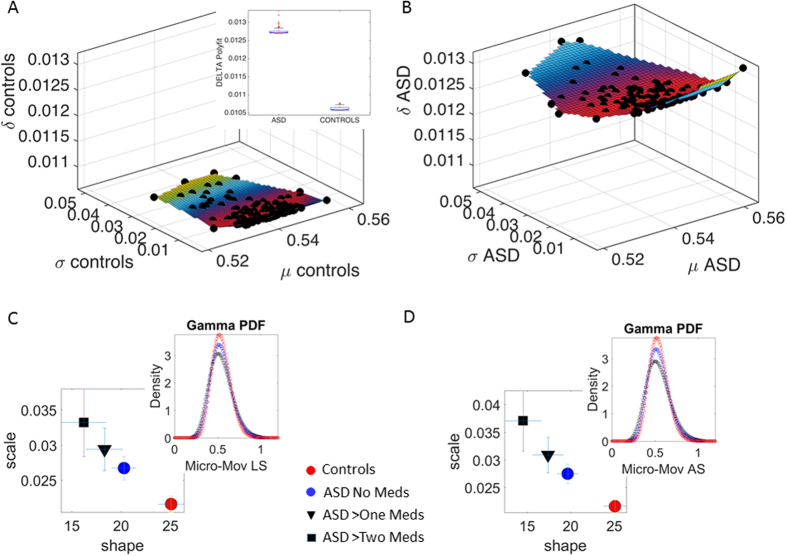
Statistical significance of the differences between ASD and control (CT) participants as a function of the number of medications reported. (**A**) Three dimensional surface fitting the parameter points of the CT and (**B**) of the ASD. Inset shows result of the Kruskall-Wallis (one way non-parametric ANOVA) test with statistically significant differences at the 0.01 level for comparison differences between the two groups on the delta residual from the polynomial fit to the scatters in A-B (ranksum Wilcoxon test, P < 10^−4^ for shape and noise, and p < 10^−41^ inset). (**C**,**D**) The role of medication status (0 *vs*. 2 *vs*. 3 medications) on the stochastic signatures of the head micro-motions (linear **D**, angular **E**) for ASD participants in relation to controls. The shape and scale parameterization of the normalized linear peak speed and normalized angular speed indexes with 95% confidence intervals show systematic shifts for individuals with ASD who are medication-naïve (no medications taken), those who take two or more, or three or more medications *vs*. controls.

The scatter was examined along three dimensions comprising the mean, the standard deviation and the delta residual as a measure of failure to follow the power law. This is shown in Fig. 4A for CT and in 4B for ASD participants. The inset in Fig. 4A shows the box plots resulting from the Kruskall-Wallis test (p < 0.01). The surface fitted to the scatter agrees with Fig. 2A showing that the subgroup with ASD had longer physical head excursions.

Because some participants with ASD were currently taking psychotropic medication whereas others were medication-naïve (here, *not* taking medication or “off” medication at the time of the scan), a possibility remained that higher levels of noise in ASD are (at least in part) due to the side effects associated with current medication intake. (Table 3 in the supplementary material lists specific medications separately for all six sites with reported medication intake, organized by medication class and associated side effects).

### The role of psychotropic medications in the empirically estimated NSR and randomness of excess involuntary micro-movements of the ASD participants

We next asked whether there is an effect of taking two or more medications (regardless of class) on patterns of micro-movement signatures. The ASD sub-groups included participants taking no medications, two medications and three medications from the UM_1 and UM_2 study-sites presented in Figs 3 and 4A,B.

Figure 4C,D shows the results of this comparison on the Gamma plane. We found systematic increase in the levels of noise along a gradient (upwards shift along the scale axis, the estimated NSR) and in randomness (leftward shift along the shape axis towards *a* = *1*, the special limiting case of the memoryless Exponential distribution) of the head micro-movements. These signatures on the Gamma parameter plane are plotted as a function of the number of medications taken by ASD participants, systematically changing with medication intake for linear displacements/translations (C) and angular rotations (D) (non-overlapping 95% CIs for the extreme cases of non-meds ASD and three-meds ASD, both far from controls). The insets in the panels 4C and 4D show the estimated Gamma PDFs for the extreme, non-overlapping cases along the gradient of Gamma parameter values.

Table 2 of the supplementary material reports the differences in empirically estimated probability distribution functions (eCDFs) of the average linear speed, another motion parameter, as a function of the medication number. This comparison is against medication-naive ASD and CT participants from all six study-sites with reported medication intake (K-S test, P < 0.01).

Albeit stochastic patterns were significantly worse for ASD participants taking multiple medications, medication-naïve participants with ASD also showed deleterious patterns relative to typically developing CT. Given the broad range of ages (6 to 50 years old) of participants we next probed parameter estimates as a function of age, taking into consideration the specific class of medication prescribed. We consider two cases below for the average speed: (1) when a medication from a given class was part of a ‘combination treatment’ (taken with other medications) and (2) when it was taken in isolation.

### The role of medication class per age group (taken with other medications)

**Figure 5:**
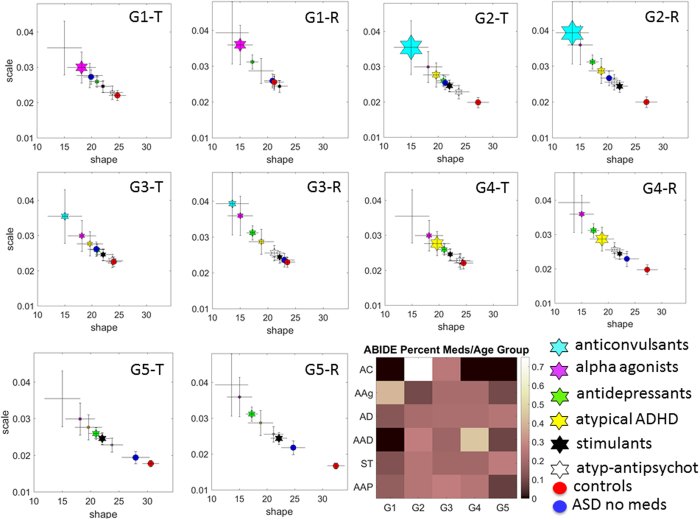
Speed-dependent stochastic signatures of head micro-movements as a function of medication status and age, for medication classes when taken as part of a combination-treatment. Each age group number and letter (e.g. G1-T and G1-R) corresponds to the linear/translational (T) and angular/rotational (R) speed-dependent signatures across medication classes shown with 95% Confidence Intervals (CIs). Groups by age are G1 (6–10.99), G2 (11–12.99), G3 (13–14.99), G3 (15–16.99), G5 (above 17) years old. The empirically estimated Gamma parameters are obtained from the pooled group data comprising ASD individuals ‘on’ medication and each point is cast against the members of age-matched reference groups (medication naïve ASD: blue and CT: red). Note that controls have the lowest NSR and the highest shape (most symmetric) value across all age groups. The size of the marker on the Gamma parameter plane represents the percentage of that medication type within the group based on the reported information in the ABIDE. The color-coded matrix presents these proportions for each age group (columns) and medication class (rows). The most commonly prescribed medication class for each age group is as follows: G1: alpha agonist, G2: anticonvulsant, G3: anticonvulsant, G4: atypical ADHD and G5: stimulant. No marker means that the medication intake is not reported in the group (matrix entry with darkest color).

Figure 5A shows parameter estimates for averaged speed in linear translations (left panel) and angular rotations (right panel) within each of the five age groups (G1–G5) for individuals with ASD and CT. Examination of 95% CIs for shape and scale parameter estimates in Fig. 5 reveals the variable differences across these different age groups between participants with ASD who were ‘on’ medication relative to both medication-naïve ASD participants and to CT. In older participants with ASD G4–G5 there is an increase in NSR across all classes of medications. In contrast younger groups show a reduction in NSR for some medications, a pattern that varies along a complex gradient from age group to age group. For example, when taken as part of a combination, the anticonvulsants, across all age groups and for both translation and rotation averaged speeds; correspond to data points that are the highest along NSR axis and the lowest along the shape axis, i.e. the farthest to the left from the CT.

The examination of the proportions of medications across age groups in the legend of Fig. 5 shows that the most prescribed medication class (in combination with other medications) is the alpha-agonist in the youngest group (G1: 6 to 10.99 year olds), whereas antidepressants and stimulants are the most prescribed in the oldest group (G5: those above 17 years old). Anticonvulsants are frequently prescribed to individuals in groups 2 and 3 (G2–G3: ranging between 11 to 14.99 years old) whereas group 4 (G4: 15 to 16.99 year olds) has the atypical ADHD medication as the most prescribed in combination with others.

Table 4 of the supplementary material reports the number of participants per class as well as the differences in eCDFs of the average linear speed (K-S test, P < 0.01, except for alpha agonists: no difference was detected between ASD participants taking alpha agonists *vs*. medication-naïve ASD participants).

### The role of medication class per age group (single medication)

We next investigated the effects of medication intake on the stochastic signatures of head micro-movements as a function of age in those ASD participants who took a single medication from a given class and no other medications.

(Table 5 of the supplementary material shows the number of ASD participants per class when medication is taken in isolation as well as the differences in eCDFs of the average linear speed (K-S test, P < 0.01, except for stimulants: no difference was detected between ASD participants taking stimulants *vs*. medication-naïve ASD participants).

**Figure 6:**
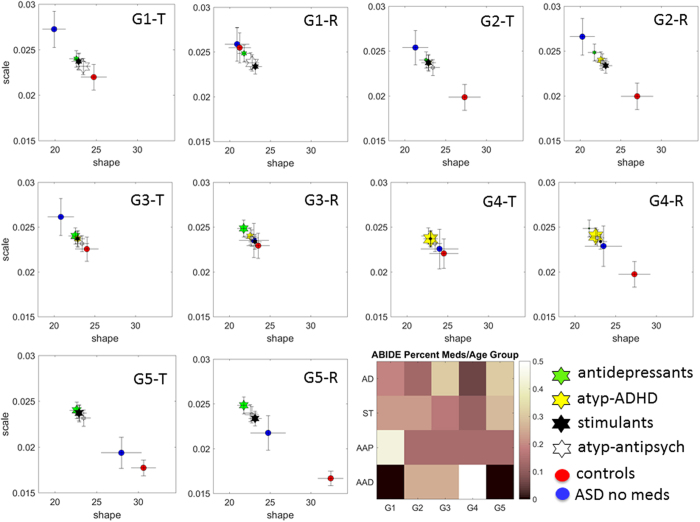
Speed-dependent stochastic signatures of head micro-motions for medication classes when taken in isolation. (Same notation as in Fig. 5; shown with 95% confidence intervals; red marker control (CT) and blue marker ASD medication naïve.) Here we considered only individuals with ASD for whom medication from a given class is prescribed in isolation, with no other medications, in relation to age-matched medication-naïve ASD participants and controls. The most commonly prescribed medication class for each age group is as follows: G1: atypical antipsychotic, G2: atypical ADHD, G3: antidepressant, G4: atypical ADHD, G5: antidepressant. Note that when the groups are comprised of ASD individuals with reported intake of a single medication class, PDFs of the same medication class (i.e., antidepressants, atypical ADHD, stimulants, and atypical antipsychotics) differ from PDFs when the same medication class is part of a combination treatment, as in Fig. 5.

Figure 6 shows the results across the five age groups (note that some medication classes are missing because fewer participants with ASD per age group were available for this analysis).

Here we see a trend of age whereby the signatures of younger ASD individuals who are ‘on’ medication are further away from those of medication-naïve ASD participants, closer towards the CT participants, suggesting a benefit (non-overlapping 95% CIs). In groups with older participants this trend is reversed. Specifically, the older the participant with ASD is, the more deleterious the role of medication status (‘on’ medication) seems to be on the NSR. This pattern consistently reveals higher NSR of the speed-dependent parameters and increased distance on their locations on the Gamma parameter plane, i.e. away from the more symmetric (Gaussian-like) distributions and lower NSR manifested by age-matched CT participants.

With regard to proportion of specific medication classes prescribed across age groups in these data bases, we found that the atypical antipsychotics are likely to be the most prescribed class in the youngest group while a higher percentage of antidepressants and stimulants are prescribed in the oldest group (congruent with the analyses above; see legend of Fig. 6).

In summary, relative to controls of comparable age, there were marked statistical differences between medication naïve ASD and on-medication ASD with visible effects that varied with age along a gradient. At the extremes of this gradient are the youngest children on-meds ASD (6-10.99 years old) who show patterns closer to age-matched controls than to meds-naïve ASD children. In contrast, the oldest group 17 years old and above show different trends whereby on-meds ASD are statistically farther apart from age matched controls than meds-naïve ASD. As the groups increase in age, the on-meds ASD group tends to shift on the Gamma parameter plane away from the corresponding age-matched controls (with variations in translation and rotation parameters).

### Influence of comorbidity and medication intake on the stochastic signatures of involuntary head micro-movements in ASD: The specific case of ADHD

**Figure 7:**
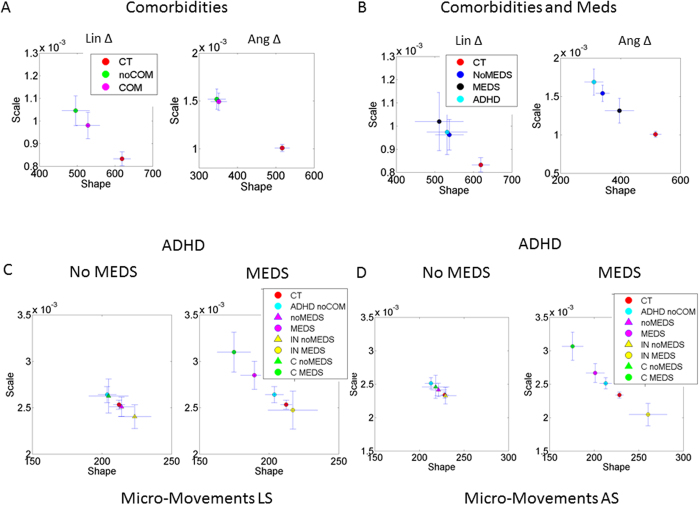
The role of secondary diagnosis (presence of comorbidities) and medication status in the signatures of micro-movements. (**A**) For ASD subgroups with (“COM”) or without (“noCOM”) comorbidities, the probability distributions empirically estimated from the fluctuations in involuntary head micro-motions fall in a region of the Gamma parameter plane that indicates higher noise and more skewed shapes for both the linear displacements and the angular rotations relative to control (CT) group. (**B**) ASD participants with a secondary diagnosis grouped by medication status (“MEDS” and “NoMEDS”). Also shown are PDFs from a subset of ASD individuals with a specific comorbidity, ADHD. Individuals with ASD who have a secondary diagnosis of ADHD have more deleterious stochastic signatures than controls. (**C**) The results from analyzing the rates of head’s linear displacements in 97 individuals with ADHD (whose diagnosis was not comorbid with ASD) from the ADHD-200 database. These signatures reveal that dissimilarities from controls increase with medication intake. In particular the signatures from participants with inattentive and combined subtypes of ADHD reveal the sensitivity of these metrics to medication intake. (**D**) Same format as in (**C**) presenting consistent results for the rates of change of the head’s angular rotations. Note that in (**C**,**D**), PDFs are shown for different subgroupings of the same cohort of ADHD participants in order to facilitate comparison to healthy controls.

Individuals with ASD often receive a secondary psychiatric diagnosis in addition to the primary diagnosis of ASD. Only one ABIDE site used in the present study, NYU, reported whether or not participants with ASD also had a comorbid diagnosis (e.g., a generalized anxiety disorder, phobia, mood Not Otherwise Specified and Attention Deficit Hyperactivity Disorder, ADHD). Figure 7A presents the empirically estimated stochastic signatures corresponding to the micro-movements (LS left and AS right) for subgroups of individuals with ASD with a reported comorbidity as well as those that do not have a secondary diagnosis, relative to the CT group. We found that regardless of the presence or absence of comorbidities, the probability distributions empirically estimated from the involuntary head micro-movements in these ASD subgroups are characterized by higher NSR and more skewed distributions as compared to those of CT controls (non-overlapping 95% CIs). Furthermore, examining ASD sub-groups comprised of individuals with a secondary diagnosis who were either “on” or “off” medication, we again found significantly noisier and more random signatures of the empirically estimated PDFs characterizing involuntary head micro-motions relative to the CT group (Fig. 7B).

Only one of the reported comorbidities, ADHD, had enough participants to permit group analysis. In particular, Fig. 7B shows high NSR levels (in the angular speed) in the signatures of individuals whose ASD diagnosis was comorbid with ADHD. (Note that the ASD subgroups were comprised of individuals “on” or “off” medication with various reported comorbidities including ADHD; a subset of these participants (with ADHD in particular) is also presented for comparison). This finding prompted us to examine datasets in the ADHD-200 database of individuals who have received a primary diagnosis of ADHD but no other secondary psychiatric diagnosis.

The investigation on the crosstalk between comorbidities and medication intake in ADHD revealed new results shown in Fig. 7C (rates of linear displacements) and Fig. 7D (rates of angular rotations). (Note that this figure presents data by different subgroups, resulting in participant overlap; this is done to facilitate comparison relative to the CT group). First, we found that overall the ADHD group (i.e., individuals whose primary diagnosis is ADHD and who did not have an accompanying comorbidity of ASD at the time of diagnosis) has different probability distributions than controls. Specifically, the empirically estimated distributions of the ADHD group are characterized by higher dispersion and more skewed shapes than those of CT. Significant differences were also found when considering ADHD subgroups comprised of those participants currently taking psychotropic medication relative to the CT group (95% CIs); no difference was detected between medication-naive ADHD relative to CT group.

We next examined signatures according to the different ADHD subtypes reported: ADHD “inattentive” and “combined” subtypes (we note that few “hyperactive/impulsive” subtype datasets were available). The inattentive subtype group shows generally atypically *lower* levels of NSR with a disparate effect of medication intake on the linear and angular speed of the head’s involuntary fluctuations (however, note non-overlapping 95% CIs only for ADHD subgroup ‘on’ medication *vs*. CT). In the linear case the medications pull the signatures towards the typical regimes, but the opposite effect is quantified in the rotations. Furthermore, among those participants currently on psychotropic medication, the ADHD “combined” subtype group shows a significant increase in the NSR and a shift in the shape of the probability distribution towards more skewed levels relative to both CT and ADHD ‘inattentive’ group (non-overlapping 95% CIs).

### Influence of ADOS clinical scores, gender, and IQ on the stochastic signatures of involuntary head micro-movements

An important question in these analyses is whether, in addition to the sensitivity of these estimated micro-movements’ stochastic signatures to medication intake and comorbidities, the signatures would also be sensitive to differences on conventional (clinical) behavioural measures. This question is challenging because clinical measures are not standardized between 0–1 values as the normalized micro-movements waveforms are. Furthermore, micro-movements are a continuous waveform providing empirically estimated stochastic signatures of a continuous random process underlying the time-series of amplitude changes in head motions. The statistical estimation process employed by this paper does not assume normality and linearity in the data. In contrast, clinical scores based on ADOS administration provided in the ABIDE are discrete and assume normality and linearity in the data. Further, IQ estimates derive from tests adapted for different age groups (i.e., via administration of child- or adult-specific tests), they provide discrete values along a standardized scale of absolute scores that do not consider the non-linear dynamical process of human physiological development (including motor control and physical growth). To be precise, age at the time of the test should be factored into the score to capture derivative (incremental) changes in scores over time. When this is done (e.g. Supplementary Figure 8 illustrating the case of IQ) the corresponding distributions of scores across the data base are not normal and the random processes under examination are not (Gaussian) as these scoring systems assume. As such, it would be inappropriate to attempt to “correlate” these sets of discrete clinical scores with the empirically estimated signatures from non-Gaussian random processes assessed in the head micro-movements. The latter are reflecting as well the behaviours of non-linear dynamical complex systems. One must keep in mind that methods such as linear regression (for example) require multi-variate normality in the parameters under examination, a condition that does not hold in these data sets under consideration.

To overcome these potential issues we median-ranked the scores and used these automatic groupings to pool their underlying micro-movements waveforms^5^. Individuals with ASD were thus grouped by their ADOS scores, ranked above and below the median values and compared to CT. Social affect (SA), repetitive and restrictive behaviours (RRB) scores, and Severity Scores (SS) were based on research-reliable administration of ADOS (scores reported were computed based on revised Gotham algorithms^25^,^26^; see Methods for details).

**Figure 8:**
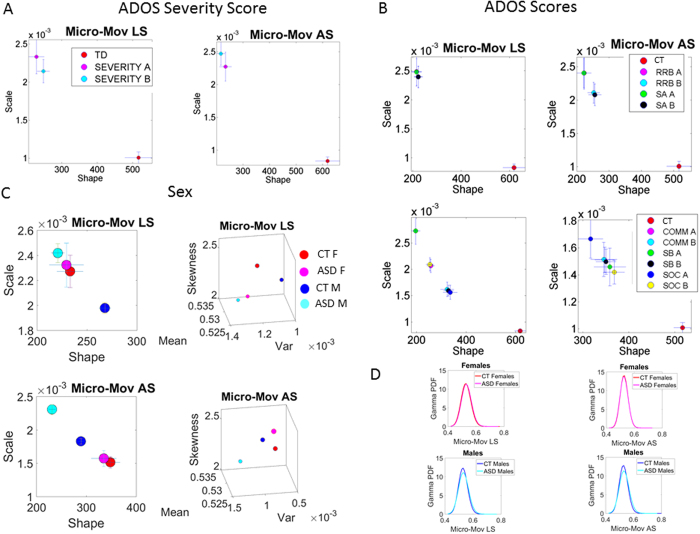
The role of ADOS severity and domain scores, and sex in the signatures of micro-movements. Behavioral and clinical scores were median-ranked; in (**A**–**D**), legend entries ending with letter “A” indicate “above” the median subgroupings while those ending with “B” indicate “below” the median subgroupings. (**A**,**B**) Higher severity scores show higher noise and higher skewness (i.e., towards the random, *memoryless* Exponential distribution) than controls. (**B**) ADOS sub-scores (Gotham algorithm was (top panel) and was not (bottom panel) available). Top: ASD subgroups with higher (“worse”) social affect (SA A) and lower (“better”) (SA B) ADOS domain sub-scores, as well as with higher (“worse”) on repetitive and restricted behaviors (RRB A) and lower (“better”) (RRB B) domain sub-scores. Bottom: ASD subgroups with communication, COMM sub-scores (“worse”: “COMM A” or “better”: “COMM B”), stereotypical behaviors, SB sub-scores (“worse”: SB A or “better”: SB B), and social communication, SOC sub-scores (“worse”: SOC A or “better”: SOC B). (**C**) Sex comparison of estimated Gamma parameters with 95% CIs reveal differences between ASD and control males. Right panels show that ASD and CT females separate in the summary statistics parameter space which plots the mean, the variance, the skewness and kurtosis of each group. Notice that the skewness separates females with ASD from control females in both LS and AS (i.e., despite their overlap on the Gamma parameter plane in the left panel). (**D**) Estimated Gamma PDFs corresponding to the estimated Gamma parameters in (**C**).

The signatures of involuntary head micro-movements grouping individuals above- and below- scores’ median ranking criteria, based on individual participants’ ADOS-SS, are shown in Fig. 8A. In the linear displacement case, the higher the severity, the higher the estimated NSR ratios in the micro-movements and the more skewed the distributions. In the rotational case an opposite pattern was found (note the overlapping 95% CIs). Both cases, however, were consistent with our main result: ASD individuals had estimated PDFs with estimated moments that indicated higher levels of dispersion and shapes significantly further away from the symmetric ones found in CT (non-overlapping 95% CIs).

Further analyses of social affect (SA) and repetitive and restrictive behaviours (RRB) ADOS scores confirmed systematic differences between ASD and CT groups. Specifically, regardless of whether the score was above or below the median, all participants with ASD were worse off than controls. Figure 8B shows that individuals in the “above-median” SA group (higher scores indicative of higher social deficits as measured by the ADOS) had the highest levels of NSR and were farthest away from the more desirable symmetric shapes in the distributions in the rotational case (non-overlapping 95% CIs). For individuals for whom revised algorithm scores were not available, we used the three scores defining communicative abilities (COMM), stereotypic behaviours (SB) and social behaviours (SOC). Consistent in all cases the NSR of ASD are elevated and the shapes more skewed in relation to CT.

Given the disparity in the diagnosis of females relative to males (approximately 5 Males to 1 Female ratio refs 27,28), we next examined stochastic signatures of females relative to males. Figure 8C shows that the deleterious effects on the probability signatures of these involuntary head micro-movements were dissociable between females and males. While the 95% CIs of the estimated signatures ASD females overlapped with those of CT females (but see below), there were significant differences between males with ASD and control males, with autistic males’ signatures in the noisiest (i.e., upper left corner) location on the Gamma plane. Furthermore, the estimated signatures of males are noisier and more random relative to females.

This exploratory analysis indicates that our overall between-group differences may be driven mostly by the differences in PDF parameter estimates in males; this finding should be considered preliminary and requires future investigation with larger female samples. Nevertheless, note that although in the current work we include both males and females in the ASD and CT cohorts, exclusion of female participants would only strengthen significant distinction between ASD and CT participants.

Further analyses of the (excess) skewness and kurtosis parameters in these groups revealed a separation between males and females with ASD and males and females controls. The plots accompanying Fig. 8C (right panel) of the Gamma parameter plane signatures are summary Gamma statistics showing more symmetric (closer to skewness value of 3) in controls and further providing another layer of informative parameters showing differences between these participants. These differences in skewness and kurtosis are more marked in the LS component than in the AS component of the motions and reveal differences between females with and without ASD (Fig. 8D shows corresponding PDFs for these subgroups).

**Figure 9:**
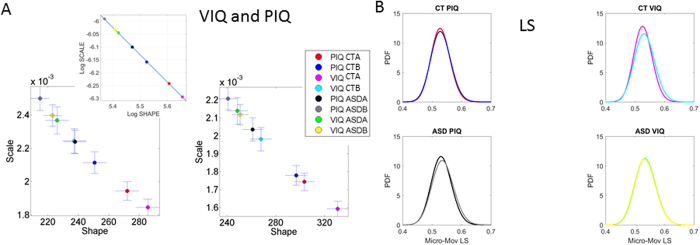
The role of IQ in the signatures of micro-movements. (**A**) Intelligence Quotient Verbal (VIQ) and Performance (PIQ) scores and stochastic signatures of all participants according to a power law (inset and see text). Participants with an IQ-score below the median had higher NSR and more skewed PDFs. Higher NSR found in ASD relative to controls (CT). Note above-median ranked PIQ scores denote “better” ability (PIQ CTA, PIQ ASDA), and below-median denote “worse” ability (PIQ CTB, PIQ ASDB); similar notation for above- and below-median ranked VIQ scores (above: “better”: VIQ CTA, VIQ ASDA; below: “worse”: VIQ CTB, VIQ ASDB). (**B**) Empirically estimated PDFs illustrate the narrow signal bandwidth in ASD and the higher NSR than CT across VIQ and PIQ in contrast to controls. Specifically, the range of VIQ PDFs for CT is much wider than the range corresponding to the PIQ. In contrast, for ASD individuals this pattern is inverted: their range of PDFs is broader for the PIQ and very narrow for VIQ.

Finally we investigated the stochastic signatures of ASD and CT participants in relation to reported verbal and performance intelligence quotient (VIQ and PIQ) subscale scores, comparing groups above the median values *vs*. those below the median value for each IQ subscale. Figure 9A shows the results for the LS (left panel) and AS (right panel). Here we report an emergent power-law relation in the inset graph between the median-ranked IQ scores and the log-log of the empirically estimated stochastic signatures for the rate of change in linear displacements and angular displacements respectively: *f(x*) = *p*_1_*x* + *p*_2_ where the coefficients (with 95% confidence bounds) are *p*_1_ = −1.06 (−1.07, −1.04), with goodness of fit: SSE: 1.304e-05, R-square: 0.9998, Adjusted R-square: 0.9998 and RMSE: 0.001474 and *f(x*) = *p*_1_*x* + *p*_2_ where the coefficients (with 95% confidence bounds) are 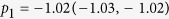, 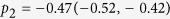 with goodness of fit: SSE: 6.42-06, R-square: 0.9999, Adjusted R-square: 0.9999 and RMSE: 0.001034.

Given the *empirically estimated* Gamma shape and NSR of the PDF characterizing the involuntary micro-movements, this power law predicts the level of VIQ and PIQ that an individual with ASD most likely has in relation to a typically developing CT individual.

This power law holds across the cross-sectional data of this population at large, i.e. including ASD and CT participants. Specifically this relation predicts that the higher IQs are characterized by lower NSR of head micro-movements and more symmetric shapes of the PDFs. In opposite fashion, lower IQs are characterized by higher estimated NSR and highly skewed distributions tending towards the limiting case of the memoryless Exponential range. Finally, note that in the case of typically developing controls, the range of estimated parameters in the VIQ domain is much broader than those in the PIQ domain. This pattern is inverted in ASD. Their PDFs show a broader range in the PIQ than in the VIQ domain (Fig. 9B).

## Discussion

We have shown that when asked to remain still, the stochastic signatures of involuntary head fluctuations captured by the micro-movements of ASD participants rapidly and randomly accumulate noise relative to the age- and sex-matched CT participants across each of the seven ABIDE study-sites. Significantly higher noise-to-signal levels are found in medication-naïve ASD individuals; these signatures are systematically worse for those with reported intake of psychotropic medication (Fig. 5). We also found an interaction between stochastic signatures, medication class and age. In particular, when a drug from a given class was taken in isolation (i.e., not as part of a combination treatment), ASD participants in younger age groups showed stochastic patterns that were closer to normative patterns of age- and sex-matched controls. In older participants an opposite effect was detected (Fig. 6).

As the individual with ASD continuously moves, however slightly, the moment-by-moment kinesthetic feedback signal (i.e., stemming from the self-generated motor output, registered by the proprioceptors, and echoed back to the CNS) has high uncertainty, contributed by excess noise and randomness in the involuntary head micro-movements. The power law relation unveiled by this work quantifies the likelihood of having lower motor NSR and more symmetric distributions with higher verbal and performance IQ scores in general—for both typically developing CT and ASD individuals (Fig. 9A inset). In ASD, the range of PDFs (e.g., for VIQ) was much narrower compared to CT participants (as shown in Fig. 9B) but interestingly their PIQ showed broader ranges than their VIQ.

For individuals with ASD, the power relation also revealed that higher social deficits (as characterized by worse ADOS SA scores) correspond to atypically higher motor noise accumulation rates and atypically more skewed PDF shapes derived from these spontaneous motor fluctuations (Fig. 9A). This is to the best of our knowledge the first time that *discrete* subjective clinical and IQ scales have been related through a lawful relation to *continuous* physical objective scales without assuming (or imposing a priori) normality on the physiological data. This is noteworthy because standardization permits comparison of individuals with heterogeneous neurological and neuropsychiatric disorders with different degrees of severity relative to typically developing individuals across the human lifespan^3^. This is arguably an important step in implementing the Precision Medicine approach in Psychiatry as it allows for integration of knowledge across different and complementary levels of information, namely those pertaining to behavioural descriptors derived through interpretation of observation and descriptors that represent actual physical measurements underlying those behaviours.

These observations have important ramifications. Although we found higher noise levels in individuals with worse social/communication scores on the ADOS instrument, we caution against the notion that individuals with ASD may lack the will or ability to form or use intentional thoughts^29^,^30^,^31^. This is because the intention to act *vs*. the volitional control over the intended act are two dissociable aspects of the human experience^32^. Because prediction and action are continuously mediated by sensory motor feedback, one important consideration is that while the specific structure of perceptual experience (e.g., a prevalence of spontaneous random noise in the returning motor signal *vs*. a prevalence of well-structured systematic noise giving rise to detectable signals) would constrain performance aspects of observable behavior, it would not necessarily reveal an individual’s intrinsic limit (underlying competence) with regard to intelligent and social functioning. The extent to which excess noise accumulation at the *involuntary* level may interfere with socio-motor behavior warrants future inquiry.

Although in the present study we used existing expert clinical classification as the basis for performing our comparisons, the same methods could be used to blindly identify self-emerging clusters in a large group of individuals of varied age, medication intake, IQ, and other characteristics (e.g., environmental exposures) as a function of interaction between these factors and the rates of change in stochastic signatures across different individuals who may or may not have clinically diagnosed pathology. Put simply, because the degree of noise accumulation is presented on a continuous, normalized scale, one can classify individuals according to where they fall in relation to other individuals. In particular, by first characterizing typical levels of NSR, we can identify atypical patterns. Here we found that participants with ASD and those with ADHD “combined” type had higher levels of noise in contrast to individuals with the “inattentive” type of ADHD, who had atypically *lower* levels of noise relative to control participants (Fig. 7C,D).

Given these results, the biologically-informed core feature representing the degree of noise accumulation during spontaneous head micro-movements in individuals with ASD may constitute a new dimension within the Research Domain Criteria (RDoC) framework that cuts across research domains^33^. Here we suggest that the current data from 1048 individuals provide a strong case for this new dimension. The individuals with neurodevelopmental disorders such as ASD have a coping nervous system that evolves in ways unique to the person. Unfortunately the current model of diagnosis fails to account for these coping capabilities of humans. In the context of neurodevelopment, this is particularly pertinent since the developmental rates of physical growth are in a non-linear accelerated state of change^34^. The methods presented here may capture precisely the non-stationary, stochastic nature of the dynamic motor phenomena underlying natural behaviours in a system characterized by non-linear relations impacting the degrees of variability^35^ across the various levels of motor control^2^. Hence, the methods and the problem at hand are congruent with each other.

Overall, the non-Gaussian nature of motion data parameters underlying natural behaviours indicates that traditional analytic approaches that assume normality, average or smooth out nuisance patterns in the kinematics data may instead discard biologically valid signals. Therefore, our findings pave the way towards new choice of analytical techniques to be used in autism research, research involving clinical trials of drugs approved for other disorders and more generally for drug development using animal models. In particular, owing to its sensitivity to changes in motor output, this unifying statistical approach can be used in drug development and testing across the different clinical Phases. For example, it can be used for more precise characterization and continuous tracking of behaviours in (transgenic) animal models (e.g., CRISPR), now including primates. It may also allow for more precise ascertainment of individual-specific side effects during human testing in Phase I of clinical trials as well as to reveal more individually-specific information in the final phase (e.g., in clinical trials involving individuals with neurodevelopmental disorders).

Because it is the nature of stochasticity and noise accumulation rates, and not the magnitude of movements over the fixed scan *per se* that is the focus of the current work, we note that eliminating the scans with higher values of the head motions may not entirely resolve the problem of movement-related signal degradation in imaging research^14^,^36^. Individuals affected by ASD physically move more and accumulate noise in a non-linear manner relative to CT (Fig. 2A) over the course of the scan. The precise role of non-linear noise accumulation from actual physical motions (that incur in energy consumption) on image quality and inference requires future investigation.

One limitation of the current study is unavailability of medication dosage, as well as treatment duration, of the medications taken by participants with ASD. The potential presence of outliers from a particular drug class may have somewhat affected the findings, presenting the possibility that those participants with significantly higher doses and those taking medication longer-term might show worse sensory-motor patterns. Note that this possibility would not alter the main findings of the study, given the tight confidence intervals characterizing CT relative to ASD participants regardless of medication status. Nevertheless, it remains to be demonstrated if changes in head micro-movements directly capture targeted changes in symptomology brought about by a specific medication. Examination of other neurodevelopmental disorders often comorbid with ASD, including ADHD, also contributed to our understanding of the influence of medication intake and its potential impact on stochastic signatures of spontaneous fluctuations of movements (Fig. 7C,D).

Finally, our goal was to harness available phenotypical and demographic data in order to link these to the character of stochastic signatures of involuntary head micro-movements in ASD and CT individuals. Relatively coarse temporal resolution of fMRI afforded fewer peaks’ fluctuations per individual participant over the course of the scan than the desirable amount required for statistical power in the empirical estimation analyses carried on here. Hence, we needed to pool the standardized micro-movements data from many individuals in order to derive reliable PDF estimates in these subgroup analyses. Nevertheless, this work represents the largest effort to date in linking phenotypical and physiological information during simple resting behavior. No matter which features we used when forming subgroups of ASD and CT participants, we found that stochastic patterns in ASD participants were furthest away from the normative data estimated from CT participants, including the ADHD ‘inattentive’ group with atypically lower levels of NSR.

The results from these analyses demonstrate the extreme usefulness of Big Data made publicly available to the scientific community, raising the question of whether the observable manifestations of autism (i.e., clinical ASD-symptomology as conventionally conceived) are fundamentally driven by systemic, *increased* noise levels in affected individuals. Data from 1048 individuals establish the specificity of this biophysically informed feature of ASD. It is also clear that the non-linear, dynamic nature of the developing mind and brain implies that potential deviations from typical development cannot be meaningfully captured with static, discrete, and linear parametric scales a priori imposed. Combined with analytic approaches that target the empirical estimation of individual variability, this information may pave the way towards a transformative path in the conceptualization and integration of information across multiple levels of the knowledge network to improve dimensional classification, diagnosis, treatment and tracking of mental illnesses, in line with the dictums of Precision Medicine^1^. Our findings seem key to more than one line of future inquiries at the intersection of personalized neuroimaging research, the personalized assessments of pharmacological treatments, as well as classification, detection and objective profiling of disorders of sensory-motor noise across neurodevelopment and beyond.

## Materials and Methods

### Experimental Design

Datasets used in this study were obtained from public, freely accessible Autism Brain Imaging Data Exchange (ABIDE) database (http://fcon_1000.projects.nitrc.org/indi/abide/). Data are de-identified in compliance with U.S. Health Insurance Portability and Accountability Act (HIPAA) guidelines. Participants at all sites signed written informed consent and assent (and parental consent, if participants were less than 18 years) in accordance with U.S. 45 CFR 46 and Declaration of Helsinki for participation; research protocols which included neuroimaging and clinical assessments at each site, were approved by the local ethics committees. Analyses of these de-identified data were reviewed and approved by Institutional Review Boards of Rutgers University and Columbia University Medical Center.

### Inclusion/exclusion criteria

In the current study, ABIDE sites were included that (*i*) deposited raw resting-state functional Magnetic Resonance Imaging (MRI) scans (i.e., no motion correction or “scrubbing”^14^ was applied to these data), (*ii*) had a total scan duration at least 8 minutes and/or (*iii*) had at least 15 individuals with ASD. Seven of 16 ABIDE sites met these overall inclusion criteria: University of Michigan, Sample 1 and Sample 2 (“UM_1” and “UM_2”, respectively), University of Utah School of Medicine (“USM”), New York University Langone Medical Center (“NYU”), University of California, Los Angeles, Sample 1 (“UCLA_1”), Olin, Institute of Living at Hartford Hospital (“OLIN”), and University of Pittsburgh School of Medicine (“PITT”).

Full batteries of non-parametric (distributional) analyses were performed on datasets from the 3 sites that met criteria (*i*) and (*ii*), UM_1, UM_2, and USM. A complementary set of analyses, reported in the Supplementary Results and in the last two figures of the main text was performed for the four remaining sites (NYU, UCLA_1, OLIN, and PITT) that deposited raw data with a shorter total scanning time (i.e., that could not be subjected to the full battery of non-parametric distributional analyses) but that had met criteria (*iii*). Note that NYU excluded individuals with the most severe head movement from the dataset but otherwise no motion correction was applied to the deposited data. All datasets deposited by each of the seven sites were analyzed.

Datasets from a total of 605 participants were analyzed, including 304 individuals with Autism Spectrum Disorder (ASD) and 301 typically developing controls (CT). Specifically, we analyzed 246 datasets from the three main sites broken down as: UM_1 (N_ASD_ = 55; N_CT_ = 55), UM_2 (N_ASD_ = 13; N_CT_ = 22), and USM (N_ASD_ = 58; N_CT_ = 43) (Total: N_ASD_ = 126, N_CT_ = 120). The rest of the sites included 178 ASD and 197 CT controls. These data break down as: NYU (N_ASD_ = 79; N_CT_ = 105), UCLA_1 (N_ASD_ = 49; N_CT_ = 33), OLIN (N_ASD_ = 20; N_CT_ = 16), and PITT (N_ASD_ = 30; N_CT_ = 27) (Total: N_ASD_ = 178; N_CT_ = 181).

Additional information on participants’ inclusion/exclusion criteria at the original ABIDE study-sites is listed in the Supplementary Material.

### Demographic characteristics

#### Main analyses datasets

Participants at the three main sites (UM_1, UM_2, USM) did not differ in age 17.51 (7.28) (mean and standard deviation; range: 8.5–50.22) for the ASD group, and 17.14 (6.22) (range: 8.2–39.39) for the CT group (p = 0.67). Participants did not differ in sex (ASD: 116/10 (Males/Females); CT: 102/18 (Males/Females) (*X*^2^ = 3.04, p = 0.08). 103 ASD participants were right-handed, 14 were left-handed, 1 was ambidextrous; 105 CT participants were right-handed and 12 were left-handed. Scores were missing for 8 ASD and 3 CT participants.

#### Additional datasets

Participants at the four other sites (NYU, OLIN, UCLA_1, PITT) also did not differ in age 15.13 (6.06) (mean and standard deviation; range: 7.13–39.1) for the ASD group, and 15.88 (5.80) (range: 6.47–33.24) for the CT group (p = 0.22). Participants did not differ in male to female ratio (ASD: 153/25 (Males/Females); CT: 145/36 (Males/Females) (*X*^2^ = 2.17, p = 0.14). 149 ASD participants were right-handed, 26 were left-handed, 2 were ambidextrous; 168 CT participants were right-handed and 8 were left-handed. Scores were missing for 1 ASD and 5 CT participants.

### Psychotropic medication intake

All sites in the current study, except USM, reported whether or not ASD participants were currently taking medications. Only one site in the current study, NYU, asked patients on stimulants to withhold their intake during the scan day. We classified reported medications into nine classes, listed in Table 3 of the Supplement along with the number of participants per class.

### Comorbidities

Only one site used in the present study, NYU, reported whether or not participants with ASD had a secondary diagnosis. Out of total participants with ASD (N = 79), N = 41 had one or more comorbidities (for example, including phobia, generalized anxiety disorder, mood Not Otherwise Specified (NOS), and ADHD) and total number per comorbidity was small, except ADHD. A total of 15 individuals with ASD also had at least one type of ADHD diagnosis (either ADHD “inattentive”, ADHD “combined”, ADHD NOS). We note that according to the most recent version of DSM (DSM5), diagnoses of ASD and ADHD are not mutually exclusive.

### Specific Instructions at each Site to participants during the resting scan

Participants at the 3 main sites (UM_1, UM_2, and USM), as well as at UCLA_1 and OLIN were asked to keep their eyes open. At NYU, most of the data were contributed from studies that asked participants to keep their eyes open during the scan, but also included data from studies that asked participants to keep their eyes closed. Participants at the Pittsburgh School of Medicine were asked to keep their eyes closed. Additional information on eye status for each site is reported at http://fcon_1000.projects.nitrc.org/indi/abide/ and also in the Supplemental Methods section.

### MRI acquisition parameters

Resting-state functional MRI (rs-fMRI) Blood Oxygenation Level Dependent (BOLD) data were acquired on GE (GE Medical Systems, Milwaukee, WI) or Siemens (Siemens Healthcare, Erlangen, Germany) 3 Tesla MR scanners. BOLD signal was obtained with T2*-weighted echo planar imaging (EPI) sequence for all of the seven sites used in the present study. All three main sites had identical inter-scan interval (repetition time = TR) of 2000 ms (½ Hz temporal resolution), and a comparable total scan duration. UM_1 and UM_2 scans were 10 minute each (300 volumes) and USM was 8 minutes (240 volumes). NYU scan was 6 minutes (180 volumes; TR = 2000 ms (½ Hz)); OLIN scan was 5 minutes 15 seconds (210 volumes; TR = 1500 ms (1/1.5 Hz)); UCLA_1 scan was 6 minutes 6 seconds (120 volumes; TR = 3000 (1/3 Hz)); PITT scan was 5 minutes 6 seconds; (200 volumes; TR = 1500 (1/1.5 Hz)). Additional details on the scanner equipment and acquisition parameters used at each site are provided in the Supplemental Methods section.

### Information on ADHD-200 database datasets

In order to investigate the issue of comorbidities more fully (given that diagnoses of ASD and ADHD are not mutually exclusive according the most recent version of DSM5), in addition to the ABIDE datasets, we also examined datasets from ADHD-200, an open-access, publicly available database (http://fcon_1000.projects.nitrc.org/indi/adhd200/) of adolescents with Attention Deficit Hyperactivity Disorder. Analyses of these de-identified data were approved by Rutgers and Columbia University Medical Center Institutional Review Boards. We included ADHD-200 study-sites that contributed several functional runs per participant (New York University Child Study Center, NYU, Oregon Health State University, OHSU) as well as a study-site with a single run (Peking University, PEKING) whose total duration was at least 8 minutes. Data in NIfTI format was downloaded and datasets from 443 participants for whom medication status was available, N_ADHD_ = 175; N_CT_ = 268 were analyzed. Out of 175 ADHD participants, 114 were not currently on medication, while 61 were on some type of psychotropic medication (none of the CT participants were currently taking psychotropic medication). Specific medication class or name was not available in ADHD-200 database. The 443 datasets from the three sites break down as: NYU (N_ADHD_ = 45; N_CT_ = 86), OHSU (N_ADHD_ = 28; N_CT_ = 39), and PEKING (N_ADHD_ = 102; N_CT_ = 143) (Total: N_ADHD_ = 175, N_CT_ = 268). Participants at the three sites (NYU, OHSU, PEKING) did not differ in age 11.40 (2.56) (mean and standard deviation; range: 7.17–17.96) for the ADHD group, and 11.60 (2.49) (range: 7.24–17.43) for the CT group (p = 0.41). rs-fMRI data were acquired at 3 T at all sites. NYU and PEKING acquired scans at TR = 2000 ms, while TR was 2500 ms at OHSU. Usable NYU datasets had 360 volumes per participant, OHSU had 246 volumes, and PEKING had 240 volumes. Additional information, including the inclusion criteria, demographic characteristics, and scanner and MRI acquisition parameters, is listed in the Supplemental Methods section.

Data from the 3 sites were pooled in order to probe the issue of comorbidities, medication intake, and ADHD clinical subtypes. N_ADHD_ = 97 had no reported comorbidities (N_ADHD_ = 61 were medication-naive, while N_ADHD_ = 36 were currently on psychotropic medication). Considering ADHD individuals with no comorbidities who were medication-naive, N_ADHD_ = 21 had ADHD “combined” subtype while N_ADHD_ = 38 had ADHD “inattentive” subtype (Considering ADHD individuals with no comorbidities who were currently on medication, there were also N_ADHD_ = 21 who had ADHD “combined” subtype and N_ADHD_ = 15 had ADHD “inattentive” subtype).

### Pre-processing of raw resting-state volume image files

Head movement parameters were obtained using Statistical Parametric Mapping (SPM8), a freely available, widely used software for processing neuroimaging data (http://www.fil.ion.ucl.ac.uk/spm/software/spm8/) running MATLAB version 8.3 (R2014a) (The MathWorks, Inc., Natick, MA).

Head movements introduce changes in signal intensity of collected volumes over time and represent a major confound in neuroimaging^37^. Thus, software for processing MRI data commonly include a motion estimation component (In SPM, the ‘realign’ component includes ‘estimate’ and ‘reslice’; the ‘reslice’ function resamples the volumes using estimated motion parameters).

In SPM, realignment of scanned volumes involves estimating the six parameters of an affine ‘rigid-body’ transformation (b-splines interpolation using least-squares approach) that minimizes the differences between each successive scan and a reference scan^20^,^37^. The default reference scan in SPM8 is the first scan (volume), to which all subsequent volumes are realigned. The output with the six motion parameters (3 translations in x, y, z directions, and 3 rotations: pitch (about x-axis), roll (about y-axis), and yaw (about z-axis)) is recorded as an rp_%s.txt file. Additional information on movement estimation is listed in the Supplemental Methods section. We separately processed raw NIfTI (.nii) files for each site in the ABIDE and ADHD-200 database used in the current study because of differences in the inter-scan interval (Repetition Time, TR), number of slices, and total scan duration (number of volumes) across sites.

### Analytics

#### Definition of micro-movements

Raw biophysical data continuously registered from physiological sensors (i.e. data derived from *physiological rhythms* such as electroencephalography, electrocardiogram, respiration patterns, kinematics from bodily, head and eye movements, tremor data, etc.) give rise to time series of spikes. The fluctuations in amplitude and timing of the spikes are assumed to characterize a continuous random process where events in the past may (or may not) accumulate evidence towards prediction of future events. The spike trains derived from such peaks (coined “the micro-movements”) are used as input to a Gamma process to empirically estimate the Gamma parameters and track their values on the Gamma parameter plane, compute the PDFs, obtain the summary statistics, etc.

More specifically, the present work assesses the scan-by-scan velocity-dependent variations in the linear displacement and in the angular rotations of the head during rs-fMRI sessions. The analyses specifically refer to the stochastic signatures of those micro-movements (as generally defined above), their accumulation and empirically-estimated statistical features. In the specific case of rs-fMRI data here, the data types used in this work are not the original head motions per se, but rather derivative information pulled out from the original time series that the head-motion extraction methods create. The commonly used methods to estimate volume-to-volume head movement from fMRI data were used here to obtain the original time series of (raw) head motion data. Then, new data types (various spike trains, e.g. peaks in speed maxima and peaks in averaged min-to-min speed of LS and AS) were derived from the time series of head motion speed. These spike trains (coined the micro-movements *waveform*) served as input to the Gamma process and a stochastic characterization of their fluctuations in amplitude used to provide a signature of the ASD *vs*. CT groups under various comparisons (sex, medication-intake status, ADOS-scores, IQ, etc.) Note that in this process the order of the amplitude peaks is preserved and the fluctuations in amplitude examined in the order in which they occurred. However, their index in the original time series is not used in this work.

Supplementary Figure 3 provides a summary of the data types used in the stochastic analyses. Further, once obtained, the micro-movements waveform can be normalized and scaled between 0 and 1 to account for allometric (head or body size) effects in cross-sectional data from the population at large. This standardized way of examining physiological signals further permits grouping of the movement data using clinical and demographic features of diverse participants.

The rate of change of linear displacement (angular rotations) was obtained in vector form (a three-dimensional velocity field over time). For each velocity vector the Euclidean norm was used to obtain the magnitude of each element in this scalar field over time, i.e. the linear speed temporal profile corresponding to the given session (denoted LS). In the cases of the angular velocity the three rotational components were Euler angles; these were converted to quaternions for proper use of the Euclidean norm on the angular velocity field. The resulting scalar field was used as the angular speed profile over the given session (denoted AS). The time-series of the LS and AS values were then plotted for each participant as a profile in time, measured (in seconds) across the length of the scanning session. Supplementary Figure S1A shows for a representative ASD participant the three-dimensional raw values for the linear and angular speeds. The bottom panels of 1 A show the linear (left) and angular (right) speed profiles. Panel 1B shows the data from a representative control.

We also pooled data across participants of a given study, grouped into ASD and controls (CT). Representative group data for the UM_1 study are shown in Fig. 2 of the main text. Notice the differences in speed magnitude between ASD (2 A) and CT (2B) participants. These data comprise 55 participants in each ASD and CT group of the UM_1 study (300 volumes per scan per each participant).

As explained in the introduction, we examined the continuous data as a Gamma process under the more general rubric of a Poisson Random Process (PRP) assuming independent and identically distributed (iid) random variables. This assumption will be relaxed in future work; but for the purposes of our examination here concerning the traditional *a priori* assumption of normality in the data, it should suffice to consider the simpler case of a point process where the distributions have various degrees of skewness, i.e. are not normal.

The fluctuations in amplitude (e.g. of speed maxima, of averaged min-to-min speeds, etc.) were gathered into a frequency histogram for each group as well as into a cumulative probability density function to contrast the two groups. These are shown in Fig. 2C along with the empirically estimated shape (*a*) and scale (*b*) parameters of the continuous Gamma family of probability distributions. The Gamma probability distribution function is given by: 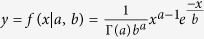, in which *a* is the shape parameter, *b* is the scale parameter, and Γ is the Gamma function^38^. We used in-house developed software and MATLAB functions to estimate the Gamma PDF using maximum likelihood estimation with 95% Confidence Intervals (CIs). Supplementary Figure 5 shows the MLE values for all 7 sites contrasting different families of probability distributions and showing the continuous Gamma family as the best fit to each data set.

The estimated parameter for each individual is plotted on the Gamma parameter plane with confidence intervals to compare the individual to others in the cohort. The data from sub-groups of participants is also pooled and the Gamma parameters estimated and plotted on the Gamma plane with confidence intervals to compare different groups in the database.

The noise-to-signal ratio (NSR), AKA the Fano Factor (FF), (Fano, 1947) is obtained from the empirically estimated Gamma variance divided by the empirically estimated Gamma mean. The Gamma mean is given by 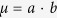 and the Gamma variance is given by 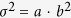. Notice that the NSR in this case is also the Gamma scale parameter since 
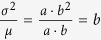
38.

This is important as we will be assessing the levels of noise in relation to the empirical estimation of the Gamma parameters from the data as a function of group type, medications, sex, comorbidities, ADOS scores and IQ across different ages. Higher levels of noise will correspond to increases of the *b scale* parameter along the vertical axes of the Gamma plane; whereas lower levels of noise will correspond to lower values along the scale axis of the Gamma plane.

It is also important to emphasize that when the *shape* parameter *a* of the Gamma family *a* = 1 the data follows the memoryless Exponential probability distribution. This is the most random distribution whereby events in the past do not accumulate information predictive of events in the future^38^. Larger values towards the right of the shape-axes on the Gamma parameter (*a*, *b*)-plane tend towards the symmetric distributions, with a variety of skewed distributions between the two extremes.

In the text we will refer to the level of randomness in the empirically estimated shape parameter (when close to *a* = 1), the limiting Exponential case (to the left); or we will point out increasing values of the shape parameter towards more symmetric distributions tending to the Gaussian limiting case (to the right). Likewise we will refer to higher or lower NSR levels according to the empirically estimated *b* Gamma scale parameter value. We will also show plots of summary statistics empirically estimated from the Gamma parameters (i.e. mean, variance, skewness and kurtosis).

Kruskall-Wallis test (non-parametric one-way analysis of variance) is used to assess the statistical significance of the differences in the empirically estimated Gamma parameters between the two diagnostic groups. Figure 2D shows the results of the median NSR compared between the two groups.

The speed maxima were normalized to avoid allometric effects due to scan length and sampling resolution differences across study-sites (Supplementary Figure S2). To this end, we obtained the averaged speed value between each two local minima in the time-series. We then divided each speed maximum by the sum of the speed maximum and the average speed between the two corresponding minima. The same procedure explained in Fig. 2 was then applied to the normalized speed maxima. Smaller values of this index indicate larger values of the average speed in the denominator (i.e. faster rates of change in linear (angular) displacements (rotations) on average). Since we are interested in the cumulative effect over time and their rates of change across the scanning session, we also obtain the empirical cumulative probability distribution function (eCDF) for these speed-dependent parameters (i.e. average speed, speed maxima and normalized speed maxima).

The empirically estimated Gamma shape and scale parameters were plotted as points on the Gamma parameter plane, each representing a study-site for the group data. In the cases where an individualized estimation procedure is performed, each point corresponds to the stochastic signatures of a single participant. In the latter case, a scatter was obtained and studied on the log-log Gamma plane in search for power law relations. The power law relation obtained is reported with the goodness of fit parameters. The fitting error between the line obtained using the estimated exponent of the power relation (the slope of the line) and the data point from the scatter was obtained for each participant and their histograms compared between ASD and controls. The Gamma scale parameter (i.e. the NSR) was plotted as a function of this error (denoted here delta) and statistical comparisons performed along each dimension. Lastly the Gamma statistics (the empirically estimated Gamma mean and Gamma variance) were plotted against the delta to fit a surface across the signatures of all ASD participants and those of the controls.

To probe the role of psychotropic medications in the level of noise in the displacement and in the rotational head micro-motions, we formed subgroups of ASD participants who were currently taking psychotropic medications, ASD participants who were medication-naïve at the time of the scan as well as CT controls. Noise analyses were conducted using participants from the two sites with the longest scan duration, UM_1 and UM_2 (note that USM did not report medication intake) as well as using pooled data from all study-sites.

We analyzed data from ASD participants (*i*) by the number of medications taken, regardless of drug class—whether they were on two or more medications or on three or more medications, (*ii*) by specific class, whether or not participants were taking this medication along with other medications—a situation which we refer to as a “combination treatment”, (*iii*), by specific class in isolation, meaning that participants took one and only one medication belonging to a given class and no other medications with it. Analyses in *i-iii* were conducted relative to medication-free ASD and CT participants. In such comparisons hundreds of speed spikes were gathered across participants per medication sub-group and the above-mentioned Gamma distributional/statistical analyses performed. These analyses were then performed as a function of age groups to elucidate interaction between medication and age. Five age-groups were identified in the data set for which a sufficient numbers of ASD participants were available per medication class to perform these statistical estimation analyses. Group1 was comprised of participants ranging between 6 and 10.99 years old. Group 2 was between 11 and 12.99 years old. Group 3 was between 13 and 14.99 years old. Group 4 was between 15 and 16.99 years old. Group 5 included all participants over 17 years old (between 17 and 50 years old).

The following classes were possible to use in the analyses where the patient was taking the medication class as part of a combination-treatment in *ii*: antidepressant, anticonvulsant, alpha agonist, atypical ADHD, atypical antipsychotic and stimulant. The following classes were possible to use in the analyses where the patient was taking the medication class in isolation, without any other medication in *iii*: antidepressant, stimulant, atypical antipsychotic, atypical ADHD medication. The results of these analyses are shown in Fig. 6, also pooling patients across all study-sites (except USM).

We also examined the relation between various clinical, demographic, and IQ scores and stochastic signatures of ASD and CT individuals. In particular, we examined individuals with ASD who received a secondary diagnosis (i.e., who had a comorbid neuropsychiatric diagnosis). In the ABIDE database, this information was confined to the NYU site. To probe the role of specificity of increased noise accumulation to ASD, rather than one or more confounding conditions as well as medication status, we also examined datasets in ADHD-200, a database of individuals who received a primary diagnosis of Attention Deficit Hyperactivity Disorder (ADHD). In addition, we examined stochastic signatures by median-ranking the clinical scores and selecting values above and below the median^5^. The scores included the reported autism severity, social and communication, and repetitive behavior on the ADOS for ASD participants as well as reported IQ for ASD and CT participants. We also examined stochastic signatures in males and females with and without ASD. The overall goal of these analyses was to confirm that the main finding of increased noise accumulation in ASD was not driven by a few individuals or by certain subgroups of individuals.

## Additional Information

**How to cite this article**: Torres, E. B. and Denisova, K. Motor noise is rich signal in autism research and pharmacological treatments. *Sci. Rep.* **6**, 37422; doi: 10.1038/srep37422 (2016).

**Publisher’s note:** Springer Nature remains neutral with regard to jurisdictional claims in published maps and institutional affiliations.

## Supplementary Material

Supplementary Information

## Figures and Tables

**Figure 1:**
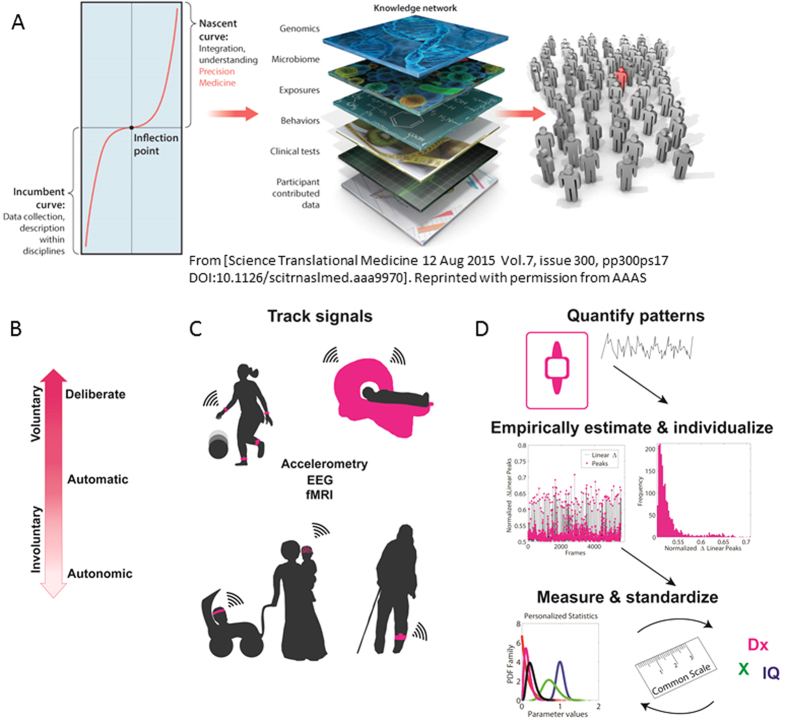
Towards true personalized medicine in Mental Health. (**A**) Poised for accelerated change in medical research and patient care using the Precision Medicine platform^1^ (From (*Science Translational Medicine* 12 Aug 2015: Vol. 7, Issue 300, pp. 300 ps17 DOI: 10.1126/scitranslmed.aaa9970) Reprinted with permission from AAAS). (**B**) Proposed taxonomy of motor-sensing-based control corresponding to different levels of variability spanning specific stochastic signatures and different ranges and families of Probability Density Functions (PDFs) across levels of neural motor control^2^. (**C**) Registration of physiological signals underlying natural behaviours is possible using a variety of devices and waveforms capturing motion generally construed as the change of position in the signal’s peaks and valleys as well as their higher order derivatives over time. (**D**) Continuous natural fluctuations in nervous systems signals are not smoothed out as superfluous noise but rather treated as spike trains reflecting random variations in amplitude and timing (the micro-movements). The stochastic signatures of these micro-movements are continuously *empirically* estimated to profile the individual using a Gamma process. The empirically estimated PDFs from this process reflect individualized rates of change in the stochastic parameters that can be mapped to a standardized scale connecting discrete clinical ratings at the bottom of the knowledge network in (A) to higher levels of the knowledge network involving continuous physiological outcomes underlying natural behaviours.
