# Supplementary material for: Motor noise is rich signal in autism research and pharmacological treatments
Source: Sci Rep. 2016 Nov 21;6:37422. doi: 10.1038/srep37422 (PMC5116649; doi:10.1038/srep37422)
Supplement: Supplementary Information [file srep37422-s1.doc]

# Supplementary Information

# Motor noise is rich signal in autism research and pharmacological treatments

# Elizabeth B. Torres and Kristina Denisova


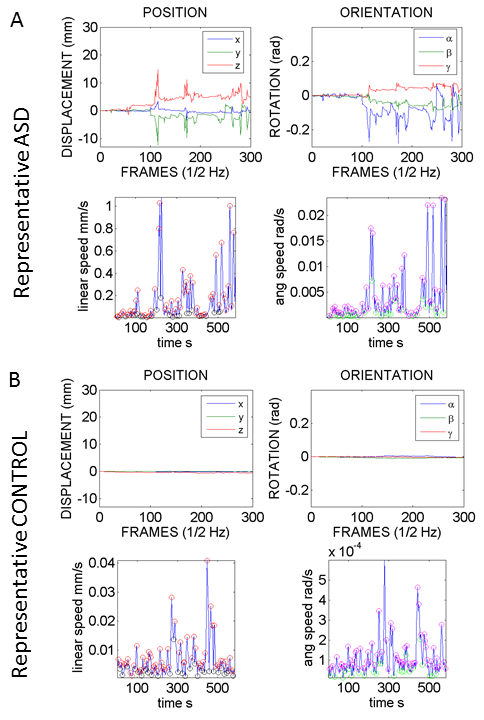


# Supplementary Figure 1

Sample raw head motions extracted from rs-fMRI data and corresponding speed profiles with kinematics landmarks of interest (peaks marked by open circles). **(A)** Displacement and rotation kinematics extracted using SPM8 from raw resting-state image files (NifTI format) provided in ABIDE (yielding 3 positional and 3 orientation parameters) . Representative ASD participant’s linear displacements and angular rotations of the head registered with respect to the first frame. Speed profiles obtained by computing the Euclidean norm of each 3 dimensional velocity vector $\left(\Delta x,\Delta y,\Delta z\right)$ displacement at each point of application $\left(x,y,z\right)$ from frame to frame, for 300 frames $speed_{frame}=\sqrt{\left(\Delta x\right)^{2}+\left(\Delta y\right)^{2}+\left(\Delta z\right)^{2}}$. To obtain velocity vector fields with corresponding speed scalar temporal profiles, the position data was analyzed using different methods and the results compared. One method filtered position data using a triangular filter to preserve the original temporal dynamics of the first rate of change data (i.e. the original timing of the peaks) while smoothing the sharp transitions from frame to frame ^1^ (using triangular window $v'(i)=\frac{\sum_{k=-d}^{d} (v(k+i)\cdot(d+1-\left|k\right|))}{\sum_{k=-d}^{d} (d+1-\left|k\right|)}$for velocity *v* of frame *i*, *k* summation index from *–d* to *d* and testing various values of *d* e.g. up to *6*, to build a symmetrically weighted sum around the center point, frame by frame). We also used regular derivative functions in the Matlab spline toolbox to transition from position to velocity. We obtained similar results as those with the triangular filter presented in the main text. **(B)** Representative control data. Notice the differences in magnitude between these two representative participants. For clarity the speed data of the control is not plotted at the same scale of the ASD panel so as to be able to see the patterns (notice that there is a large difference in the y-axis scale from A to B).


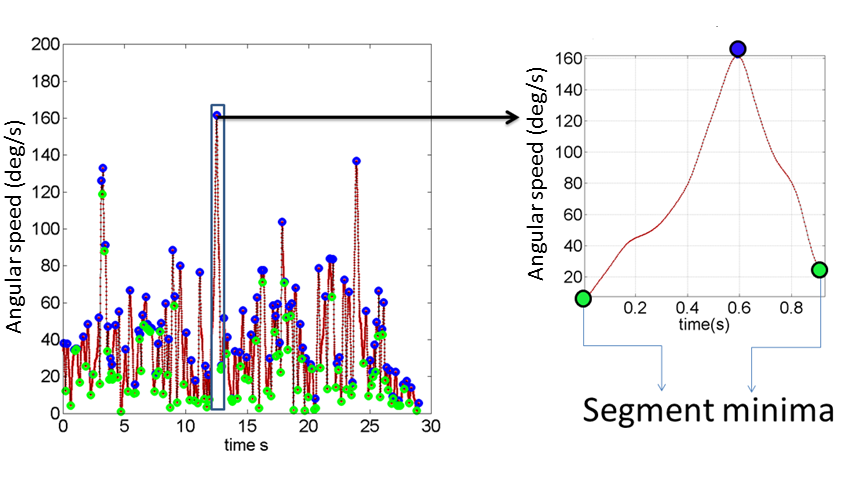


# Supplementary Figure 2

Schematics of extracting the normalized speed maxima from continuous time series of speed-dependent data. In this example (used only for explanation purposes) of angular rotations recorded with high sampling resolution sensors the speed peaks and valleys are marked by blue and green dots respectively. The zoomed-in picture of one peak between two minima is used to explain the index

$$NormSpeedMax=\frac{SpeedMax}{SpeedMax+AvrgSpeed}$$

The maximum value of the speed between two valleys (local minima) is obtained and then divided by the sum of that value and the average speed between the two minima. Higher values of average speed result in lower NormSpeedMax. Likewise, shifts towards higher values of this index indicate lower speed on average.


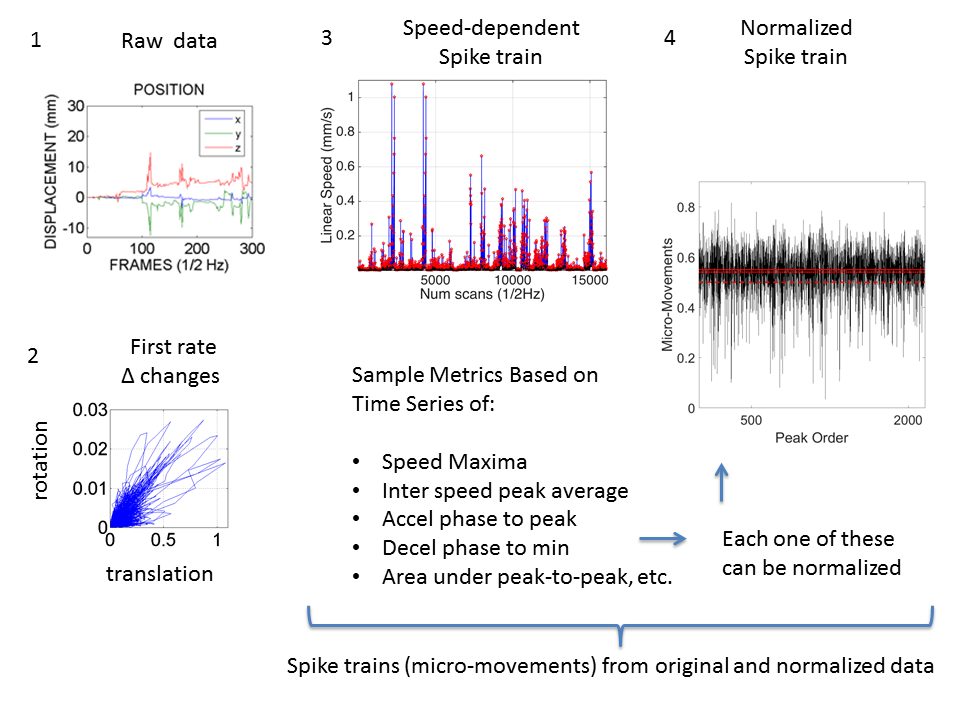


# Supplementary Figure 3

**Data processing of kinematic data**. (Step 1) Obtain raw positional data (these can be from linear displacements or angular rotations); (Step 2) Examine excursions of various types, for example first rate of change in displacements and rotations (plotted here), cumulative excursions, etc; (Step 3) Obtain the scalar (speed) magnitude the first-order rate of change time series (i.e. commonly termed velocity-dependent data). In this case the velocity obtained from positional data is computed and the scalar value (speed) obtained. The peaks are used to study their fluctuations in amplitude and timing (spike trains). Sample kinematic metrics derived from the velocity-dependent data are (among others) spike trains of speed maxima, of inter speed peak average, inter minima speed average, acceleration (rising phase) to the peak, deceleration (decay phase) to the minima, area under peak to peak, area under minima to minima, etc. These spike trains are coined micro-movements (examples of micro-movements use as input to Gamma process appear in ^2-5^; (Step 4) Normalize the micro-movements to account for allometric effects due to disparity in anatomical features in cross-sections of the population. This paper uses primarily speed-dependent micro-movements (linear LS and angular AS) and normalized versions of them.


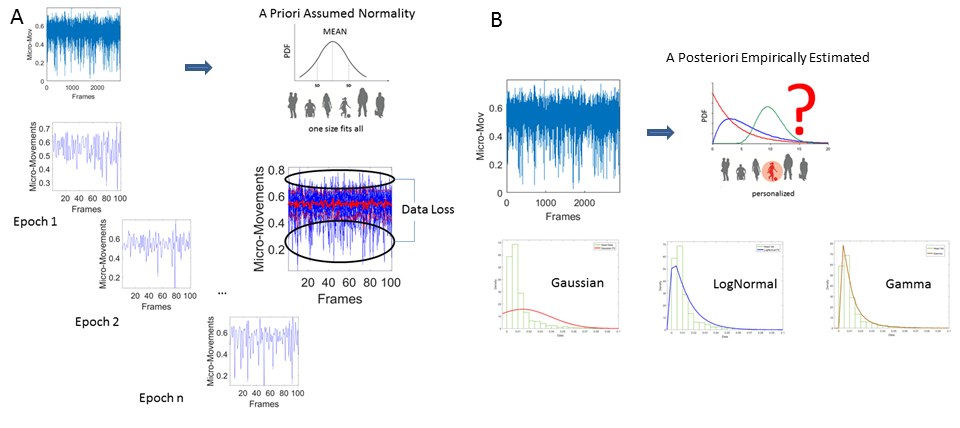


# Supplementary Figure 4

Comparison between traditional analytics and the present personalized statistical approach. (A) The standardized micro-movements extracted from the rs-fMRI head motion data pooled across the data base are scaled between 0 and 1. These spike trains represent a continuous random process modeled as a Gamma process (see text). The noise to signal ratio of these spike-events are of interest in the statistical analyses to be employed. Traditional models assume a Gaussian random process with additive statistics. As such the assumed theoretical (population) Gaussian moments (the mean and the variance) are used to process the data using the average of the peaks across a pre-set number of frames. Typically, pre-selected epochs of the data are averaged under this Gaussian population mean assumption. The sample outcome illustrating this highlights as well the data loss from smoothing out as “noise” the fluctuations beyond the red curves representing +/- the assumed standard deviation from the assumed mean. This is the traditional “one-size-fits-all” approach to data analyses. (B) The same micro-movements waveform of fluctuations in amplitude is analyzed using the approach in the present work. This does not assume *a priori* any theoretical distribution. Instead, it accumulates events till the estimation process yields tight confidence intervals for the fitting of various families of probability distribution functions. In this case the normal distribution, the lognormal distribution and the Gamma distribution are used to illustrate the process of finding the most appropriate family to characterize the data. Here maximum likelihood estimation was used with 95% confidence intervals criterion. This is the approach we have previously proposed to aid implement a true personalized method of analysis ^6^.


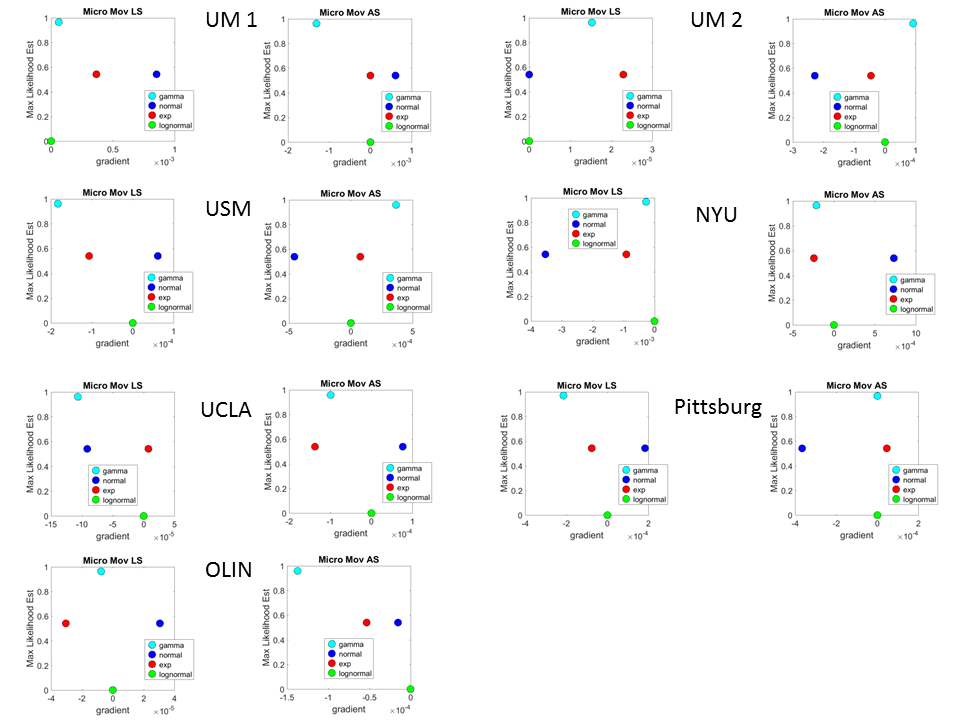


# Supplementary Figure 5

**Maximum likelihood estimated values for all 7 sites**. Each plot comprises data of all reported subjects per site (autism and controls used in the analyses.) The Horizontal axis contains the value of the gradient at the end of the optimization process (10^-6^-10^-4^ range according to the set tolerance value of 10^-30^ for the optimization process). The vertical axis contains the MLE value for the Gamma, the Normal, the Exponential and the Lognormal distributions (see legend). The respective values are: (USM, LS [0.9637 0.5409 0.5409 0.0000], AS [0.9596 0.5389 0.5389 0.0000]; UM1 LS [0.9665 0.5428 0.5428 0.0000], AS [0.9635 0.5406 0.5406 0.0000]; UM2 LS [0.9628 0.5401 0.5401 0.0000], AS [0.9628 0.5392 0.5392 0.0000]; OLIN LS [0.9635 0.5420 0.5420 0.0000], AS [0.9601 0.5404 0.5404 0.0000]; NYU LS [0.9687 0.5422 0.5422 0.0000], AS [0.9652 0.5386 0.5386 0.0000]; UCLA LS [0.9620 0.5416 0.5416 0.0000], AS [0.9590 0.5395 0.5395 0.0000]; Pittsburg LS [0.9709 0.5431 0.5431 0.0000], AS [0.9673 0.5419 0.5419 0.00]).


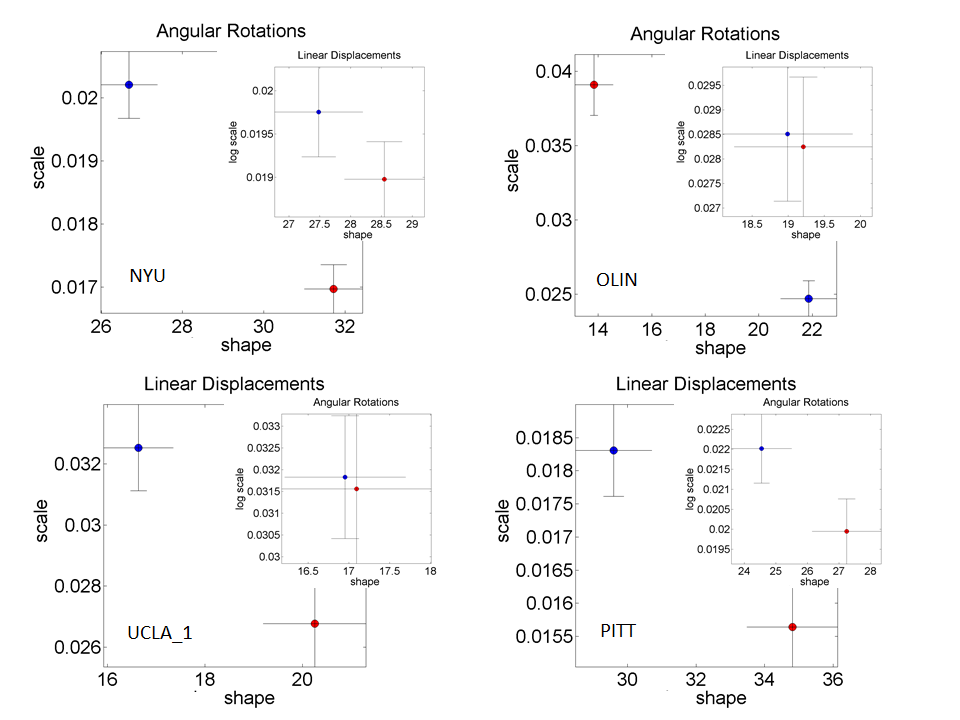


# Supplementary Figure 6

The analyses of each study site data pooled across all participants from each group (ASD and TD) showed statistical differences in the LS and AS micro-movements. Red color designates CT and blue designates ASD participants’ data. Larger plots are the LS or AS maximal separation. Insets are either overlapping CI or less separation than larger plots. The empirically estimated Gamma shape and scale parameters were obtained using maximum likelihood estimation. Estimated Gamma parameters are plotted on the Gamma plane with 95% confidence intervals for each study site. The study at NYU had 105 TD and 79 ASD participants. The study at OLIN had 16 TD and 20 ASD participants. The study at UCLA_1 had 33 TD and 49 ASD participants. The study at Pittsburgh (PITT) had 27 TD and 30 ASD participants.


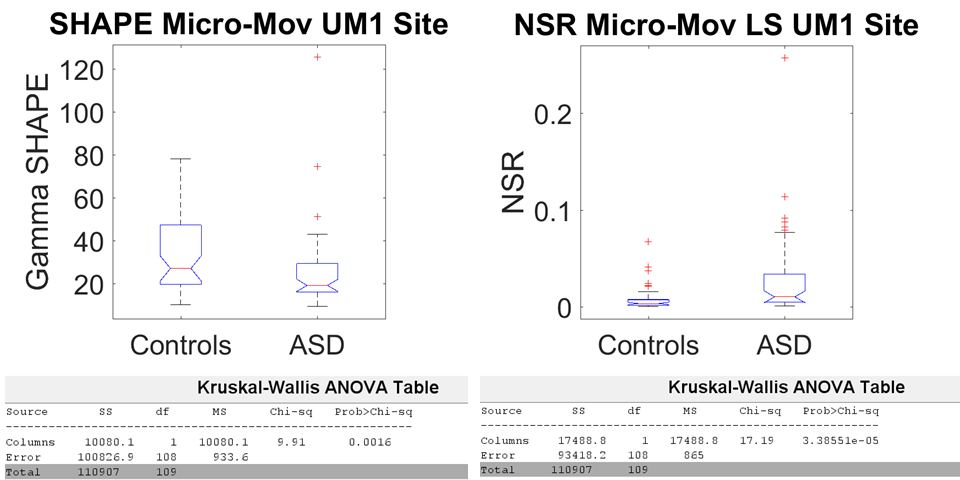


# Supplementary Figure 7

Example of statistical significant differences between ASD and controls in the shape and NSR estimated Gamma parameters.


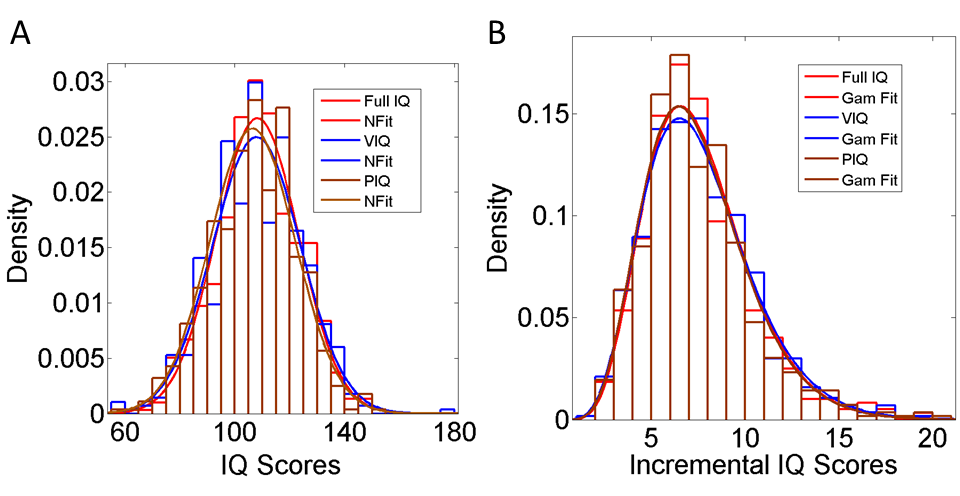


# Supplementary Figure 8

Statistical differences between absolute *vs*. incremental (age-dependent) IQ scores. (A) Frequency histograms of absolute IQ scores (full, verbal and performance) are well fit by the Gaussian distribution characterizing a random process with additive statistics. Cross-sectional data from the reported demographics in the ABIDE database includes all participants (TD and ASD ranging from 6 to 50 years old). (B) The same data corrected by the age at the time of the test spans different statistical properties. The frequency histograms of the incremental data are rather well fit by the Gamma distribution better characterizing a random process with multiplicative statistics.

**Table 1.** The results for each study-site statistical comparison of the noise levels.

| **Study Site** | **Median [range]**  **Delta error** | **Ranksum Wilcoxon** | **N participants**  **N scans/participant** |
| --- | --- | --- | --- |
| UM_1 | ASD 0.82 [0.79, 1.69]  TD 0.54 [0.52, 0.16] | $P<10^{-14}$ | ASD 55, TD 55, 300 |
| UM_2 | ASD 0.81 [0.69, 1.55]  TD 0.57 [0.54, 1.69] | $P<10^{-4}$ | ASD 13, TD 22, 300 |
| USM | ASD 0.65 [0.65, 0.68]  TD 0.46 [0.46, 0.48] | $P<10^{-17}$ | ASD 53, TD 48, 240 |

**Table 2**. Outcome of the Kolmogorov-Smirnov non parametric two-sample test on the empirically estimated cumulative distribution functions (CDFs) of the average linear speed around speed maxima taken from minima-to-minima (as in **Supplementary Figure 2** inset) when comparing 2 *vs.* 3 medication classes (1) ASD participants ‘on’ medication *vs.* TD controls; (2) ASD participants ‘on’ medication *vs.* medication-naive ASD participants; (3) medication-naive ASD participants *vs.* TD controls. Medication sub-groups are formed by participants with ASD who were ‘on’ medication, with reported intake of either 2 or more, or 3 or more medications from distinct medication classes. Note that “3 or more” group contains some participants from the “2 or more” group.

|  | Comparison Type | | |
| --- | --- | --- | --- |
| **Number of medications (ASD, N)** | **(1)** | **(2)** | **(3)** |
| Two medications (38) | 5.7494e-37 | 0.0053 | 4.3440e-65 |
| Three medications (8) | 7.2069e-24 | 0.0039 |

**Table 3.** Psychotropic medications taken by participants with ASD shown by medication class, and their reported motor and bodily related side effects.

| **Class**  **(Psychotropic medications, N)** | **Specific medications**  **(across the 7 sites)** | **Motor and Bodily Related Side Effects** | **N ***  **(Main study-sites: UM_1,UM_2)** | **N ****  **(study-sites: NYU, UCLA_1, OLIN, PITT)** |
| --- | --- | --- | --- | --- |
| Antidepressants (10) | Fluoxetine, Sertraline hydrochloride, Trazodone, Escitalopram, Citalopram, Bupropion, Mirtazapine, Duloxetine hydrochloride, Venlafaxine, Paroxetine | Tremors; paraesthesia; dizziness, drowsiness | 17 | 36 |
| Stimulants (5) | Amphetamine and Dextroamphetamine, Lisdexamfetamine, Methylphenidate Extended release, Dexmethylphenidate, Dextroamphetamine sulfate | Dizziness, drowsiness; twitching; convulsions | 14 | 30 |
| Anticonvulsants (3) | Oxcarbazepine, Valproic acid, Lamotrigine | Tremors; drowsiness | 2 | 2 |
| Atypical antipsychotics (5) | Risperidone, Ziprasidone hydrochloride, Asenapine, Quetiapine, Aripiprazole | Tremors, twitching; restlessness | 12 | 14 |
| Benzodiazepine anticonvulsant (1) | Lorazepam | Drowsiness; muscle trembling | 0 | 1 |
| Alpha agonists (2) | Guanfacine, Clonidine | Restlessness; shakiness; dizziness | 3 | 7 |
| Atypical ADHD medication (NRI) (1) | Atomoxetine | Tremors; dizziness, drowsiness | 6 | 5 |
| Nonbenzodiazepine sedative-hypnotic (1) | Eszopiclone | Clumsiness; difficulty with coordination | 0 | 1 |
| Nonbenzodiazepine anxiolytic (1) | Buspirone | Nervousness | 2 | 0 |

Note: paraesthesia is the sensation of itching, burning, numbness, prickly feeling on the skin, or the feeling of “pins and needles”. Note that these totals do not add up to N=92 (the number of ASD participants “on” medication across all sites) because some ASD participants were taking multiple medications from different classes, and some were taking multiple medications belonging to the same class. (Medication class information was missing for N=5 participants who were reported as taking psychotropic medication; these participants’ data are not part of this Table). Across the six reporting sites, participants with ASD were taking Antidepressants (N=53; includes SSRIs, NRIs, SARI and atypical antidepressants), Stimulants (N=44), Atypical antipsychotics (N=26), Anticonvulsants (N=4), Benzodiazepine anticonvulsant (N=1), alpha agonists (N=10), Atypical ADHD medication (N=10; NRI), nonbenzodiazepine sedative-hypnotic (N=1), nonbenzodiazepine anxiolytic (N=1). (None of the TD participants were on psychotropic medications, except one, whose data were excluded from medication-specific analyses). *In the main sample, there were N=11 unique participants in the ASD group on atypical antipsychotics medication (whereas N=1 was on two antipsychotic medications). Note that one study-site, USM, did not report medication intake. **In the complementary sample, there were N=28 unique participants on stimulants (N=2 were on two different stimulants). There were N=29 unique participants on antidepressants (N=5 on two antidepressant medications and N=1 on 3 antidepressant medications). Sources of reported side effects for each medication class: <https://www.nlm.nih.gov/medlineplus/druginformation.html>, [http://www.drugs.com](http://www.drugs.com/), and [http://www.medicinenet.com](http://www.medicinenet.com/).

Additional information is provided in References ^7-15^.

**Table 4**. Outcome of the Kolmogorov-Smirnov non parametric two-sample test on the empirically estimated cumulative distribution functions (eCDFs) of the average linear speed around speed maxima when comparing (1) ASD participants ‘on’ medication *vs.* TD controls; (2) ASD participants ‘on’ medication *vs.* medication-naive ASD participants; (3) 39 medication-naive ASD participants *vs.* 52 TD controls. Data for normative comparisons (N=52) are from a single site (UM_1) with the longest total scan time and the largest number of TD participants with a known medication-free status. Each medication class group is formed by participants with ASD where the medication class is composed of one or more medications in that class and is taken as part of a ‘combination treatment’.

| **Class in a combination-treatment** | **Comparison Type** | | |
| --- | --- | --- | --- |
| **Medication Class (ASD, N)** | **(1)** | **(2)** | **(3)** |
| Antidepressant (46) | 4.2786e-117 | 8.9180e-32 | 4.3440e-65 |
| Anticonvulsant ( 4) | 3.3807e-35 | 0.0088 |
| Alpha Agonist (10) | 1.4938e-21 | 0.3168 |
| Atypical ADHD (10) | 0.0027 | 2.3692e-19 |
| Atypical Antipsychotic (26) | 6.0632e-05 | 5.2869e-18 |
| Stimulant (42) | 1.0531e-37 | 4.7917e-05 |

**Table 5**. Outcome of the Kolmogorov-Smirnov non parametric two sample test on the empirically estimated cumulative distribution functions (eCDFs) of the average linear speed of the head micro-movements when comparing (1) ASD participants ‘on’ medication *vs.* TD controls; (2) ASD participants ‘on’ medication *vs.* medication-naive ASD participants; (3) medication-naive ASD participants *vs.* TD controls. Each medication class group is formed by participants with ASD where the medication class is composed of one or more medications in that class and is taken in isolation. The participant does not take other medications from a different class.

| **Class in isolation** | **Comparison Type** | | |
| --- | --- | --- | --- |
| **Medication Class (ASD, N)** | **(1)** | **(2)** | **(3)** |
| Antidepressant (16) | 4.5896e-52 | 1.1652e-13 | 4.3440e-65 |
| Atypical ADHD ( 4) | 1.2987e-05 | 1.5284e-14 |
| Atypical Antipsychotic ( 7) | 2.1210e-05 | 3.4403e-05 |
| Stimulant (18) | 1.0209e-24 | 0.1438 |

# Supplementary Methods

**ABIDE database**

**Inclusion/exclusion criteria**

Inclusion/exclusion criteria for each site are reported in detail at *http://fcon_1000.projects.nitrc.org/indi/abide/*. Each site included data for individuals with Autism Spectrum Disorders (ASD) and typically developing (TD) individuals. Note that reported criteria are similar, but are not fully uniform across the seven sites used in the current study (listed in detail below). Briefly, typically developing (TD) control participants were required to be neurologically and psychiatrically healthy individuals, ascertained on the basis of detailed health questionnaire and family history at most sites. Potential participants with Autism Spectrum Disorders (ASD) at most sites were excluded if there was a history of a neurological disorder, history of seizures, or previous head trauma with loss of consciousness. Of note, USM performed genetic testing to ensure exclusion of medical causes of autism (e.g., Fragile-X gene testing). In determining whether potential participants meet criteria for inclusion in the ASD group, all seven sites used the Autism Diagnostic Observation Schedule (ADOS-G) ^16^ and the parent interview, Autism Diagnostic Interview-Revised (ADI-R) ^17^, as well as additional criteria that varied across sites (e.g., meeting the DSM-IV-TR diagnosis of autism, Asperger’s or Pervasive Developmental Disorder Not-Otherwise-Specified (PDD-NOS)). All participants were required to have intelligence quotient (IQ) scores over 70. Sites-specific criteria are as follows.

*Exclusion criteria.* Exclusion criteria for all potential participants (ASD and TD) common to all 7 sites included contraindications to MRI (including the presence of metal implants, tattoos, pacemaker, braces, or pregnancy) and previous head trauma with loss of consciousness. In addition, potential participants were excluded at UM_1, UM_2, USM, UCLA_1, and PITT if they had any neurological disorder or history of seizures. UCLA_1 site also excluded potential participants with tic or involuntary movement disorder, or any known genetic disorder. NYU site excluded potential participants with chronic systemic medical conditions.

*Exclusion criteria for ASD participants.* In addition, reported exclusion criteria for potential ASD participants included the presence of psychosis or bipolar disorder at UM_1 and UM_2. Potential ASD participants at the NYU site were excluded if they had a manic/depressive episode, bipolar disorder, schizophrenia, or posttraumatic stress disorder, as well as if they were on antipsychotic medication. PITT excluded potential ASD participants with genetic disorder such as tuberous sclerosis or Fragile-X syndrome. At USM, exclusion criteria for potential participants with ASD were presence of medical causes of autism on the basis of history, physical exam, Fragile-X gene testing, and karyotyping.

*Inclusion criteria.* OLIN required potential ASD and TD participants to meet an intelligence quotient (IQ) cutoff of at least 70 on the full-scale IQ (FIQ), PITT required FIQ>80, USM required performance IQ (PIQ)>70, UM_2 required verbal IQ (VIQ)>=80, and UM_1 required that either VIQ or PIQ scores were >=85. Participants at UCLA_1 were reported to be required to be “fully verbal”; IQ cut-off is not indicated for NYU.

Estimates of IQ (Verbal and Performance IQ) were obtained using the Wechsler Adult Intelligence Scale (WAIS-III) ^18^ (Wechsler, 1997) at USM, using the Wechsler Abbreviated Scales of Intelligence (WASI) ^19^ (Wechsler, 1999) at UM_2, USM, NYU, UCLA_1, and PITT and using the Wechsler Intelligence Scale for Children at UCLA_1 (WISC-IV) ^20^ (Wechsler 2003). Differential Abilities Scale (DAS-II: School Age Edition)(16) was also used at UM_1. At UM_1 and UM_2, estimates were also obtained using the Ravens Standard Progressive Matrices ^21^ (Raven 1960) and the Peabody Picture Vocabulary Test (PPVT) ^22^(Dunn & Dunn 1997).

When using Ravens and the PPVT scales, which estimate performance and verbal IQ, respectively, full IQ estimate is reported as an average of PIQ and VIQ. Only full IQ estimates are reported for the OLIN site using the WAIS-III ^18^ (Wechsler, 1997) and WISC-III ^23^ (Wechsler 1991).

*Additional inclusion/exclusion site-specific criteria for TD participants.* Typically developing participants were required to be neurologically and psychiatrically healthy individuals. UM_1 excluded potential TD participants with scores > 100 on the SCQ or > 6 on the Obsessive/Compulsive Scale of the Spence Children’s Anxiety Scale (SCAS) ^24^ (Spence 1997). UM_2 screened participants for clinically-significant symptoms using the Achenbach Young Adult (Child) Behavior Checklist ^25^ , Social Responsiveness Scale-Child version ^26^ , and the Vineland Adaptive Behavior Scales-II (VABS-II)^27^ (using parental report for adolescent participants). For adults, Achenbach Behavior Checklist and the Conners rating scale ^28^ (Conners 2008) self-report forms were used. USM required that potential TD participants had no history of learning disabilities, no history of substance abuse, psychiatric disorder, and no family history of ASD in 1^st^, 2^nd^, 3^rd^ degree relatives. NYU required an absence of any Axis-I disorder, confirmed using the Schedule of Affective Disorders and Schizophrenia for Children-Present and Lifetime Version (KSADS-PL) ^29^ in children and participant interview using the Structured Clinical Interview for DSM-IV-TR Axis-I Disorders, Non-patient Edition (SCID-I/NP) ^30^ and the Adult ADHD Clinical Diagnostic Scale (ACDS) ^31^ for adults. OLIN required that ADOS-G and SCQ-Lifetime Version scores were below diagnostic cut-offs for ASD; inclusion was ascertained on the basis of a “detailed health questionnaire”. UCLA_1 required that potential TD participants “could not have a first-degree relative with ASD”. Pitt required that potential TD participants have no history of birth complications or a psychiatric disorder.

*Inclusion criteria for ASD participants.* In determining whether potential participants meet criteria for inclusion in the ASD group, all seven sites used the Autism Diagnostic Observation Schedule (ADOS-G) ^16^ and the parent interview, Autism Diagnostic Interview-Revised (ADI-R) ^17^. OLIN used either the ADI-R or the Social Communication Questionnaire (SCQ-Lifetime Version) ^32^. UM_1, UM_2, and PITT also required a “clinical consensus” or “expert clinical opinion” as the basis for inclusion of participants in the ASD group. Meeting DSM-IV-TR diagnosis of autism was required at USM, and meeting DSM-IV-TR diagnosis of autism, Asperger’s or Pervasive Developmental Disorder Not-Otherwise-Specified (PDD-NOS) was required at NYU. OLIN required that potential participants had a previous clinical diagnosis of ASD established by a medical professional unaffiliated with Olin.

*Handedness assessments across sites.* Handedness was based on either self-report (UM_1, UM_2, and OLIN), Edinburgh Handedness Inventory (Oldfield 1971) ^33^ (USM and NYU), or Annett Hand Preference Questionnaire ^34^ (PITT). Numerical scores were converted to categorical variables (Right-handed, left-handed, or ambidextrous).

## Recruitment

ASD and TD participants were recruited from community sources, using advertisements or via referrals. Specific reported recruitment details for each site are presented below. At UM_1 and UM_2, ASD participants were recruited through University of Michigan Autism and Communication Disorder Center (UMACC). TD participants were recruited from the community through flyers. At USM, ASD and TD participants were recruited from community sources (“parent support groups, youth groups, schools, social skills groups, and other organizations”). At NYU, ASD and TD participants were recruited from the New York Metropolitan area, through “flyers, magazine and we advertisements, parent support groups, referrals from the New York University Child Study Center clinical services, as well as word of mouth”. At OLIN, ASD were recruited from the Institute of Living outpatient services and psychiatric clinics in Hartford, Connecticut area. TD were recruited by word of mouth and flyers. At UCLA_1, ASD and TD were recruited from the greater Los Angeles, California area (“using flyers posted in community/youth organizations and schools, radio ads, and word of mouth”). At PITT, ASD participants were individuals with Autistic Disorder, “referred from the Center for Excellence in Autism Research (CEFAR) and the Autism Center of Excellence (ACE). TD participants were recruited from previous studies, by using flyers and announcements” in the community. (*http://fcon_1000.projects.nitrc.org/indi/abide/)*

**Eye status during the resting scan across study-sites**

UM_1 and UM_2: Participants were asked to look at a fixation cross in the middle of the screen, and were “instructed to let their minds wander and to not think about anything in particular while they looked at the cross”. USM: "Keep your eyes open and remain awake, letting thoughts pass through your mind without focusing on any particular mental activity." NYU: “Most participants were asked to relax with their eyes open, while a white cross-hair against a black background was projected on a screen. However, data were also included for some individuals who were asked to keep their eyes closed; in a few cases, participants closed their eyes regardless of instructions to maintain them open.” OLIN: “Participants were instructed to lie still with their eyes open, fixating on a centrally presented cross”. UCLA_1: Participants were asked to "Relax and think about whatever you want. Keep your eyes open and keep your head still.” A white screen with a black fixation cross in the middle of the screen was presented. PITT: Participants were “instructed to close their eyes and asked not to fall asleep.” (*http://fcon_1000.projects.nitrc.org/indi/abide/*)

## MRI acquisition parameters

Blood Oxygenation Level Dependent (BOLD) signal was obtained with T2*-weighted echo planar imaging (EPI) sequence for all of the seven sites used in the present study, with specific parameters as follows. For UM_1 and UM_2, EPI sequence parameters were: [TR (Repetition Time) /TE (Echo Time): 2000/30 ms, flip angle=90°, FOV (Field of View) =220 mm, 64 x 64 matrix, 40 contiguous axial 3 mm thick slices], with scan duration of 10 minutes (300 volumes). Slice acquisition was sequential ascending with no gap, using reverse spiral sequence (Glover and Law, 2001). For USM, EPI parameters were: [TR/TE: 2000/28 ms, flip angle=90°, FOV=220 mm, 64 x 64 matrix, 40 axial 3 mm slices], with scan duration of 8 minutes (240 volumes). Interleaved acquisition used Generalized Autocalibrating Partially Parallel Acquisition (GRAPPA) with acceleration factor = 2, .3 mm gap between slices. Prospective Acquisition CorrEction (PACE) motion correction was used. For NYU, EPI parameters were: [TR/TE: 2000/15 ms, flip angle=90°, FOV=240 mm, 80 x 80 matrix, 33 axial 4 mm slices], with scan duration of 6 minutes (180 volumes). Slice acquisition was interleaved, with no gap between slices. For OLIN, EPI parameters were: [TR/TE: 1500/27 ms, flip angle=70°, FOV=220 mm, 64 x 64 matrix, 29 axial 4 mm slices], with scan duration of 5 minutes, 15 seconds (210 volumes). Slice acquisition was interleaved, ascending, with 1 mm gap between slices. For UCLA_1, EPI parameters were: [TR/TE: 3000/28 ms, flip angle=70°, FOV=192 mm, 64 x 64 matrix, 34 axial 4 mm slices], with scan duration of 6 minutes, 6 seconds (120 volumes). Slice acquisition was interleaved with no gap between slices. For PITT, EPI parameters were: [TR/TE: 1500/25 ms, flip angle=70°, FOV=200 mm, 64 x 64 matrix, 29 axial 4 mm slices], with scan duration of 5 minutes, 6 seconds (200 volumes). Slice acquisition was interleaved with no gap between slices.

## ADHD-200 database

Open-access, freely accessible Attention Deficit and Hyperactivity Disorder (ADHD-200) database (http://fcon_1000.projects.nitrc.org/indi/adhd200/) was used to obtain datasets with resting-state data of individuals with ADHD and typically developing controls. For all ADHD-200 study-sites, participants signed assent and parental consent forms according to IRB-approved protocol procedures at each institution. Fully anonymized, de-identified datasets (free of the 18 HIPPA identifiers) were analyzed. Analysis of these de-identified data was approved by the Institutional Review Boards of Rutgers University and Columbia University Medical Center.

**Inclusion/exclusion criteria in the current study**

ADHD-200 study sites included the New York University Child Study Center (“NYU”), Oregon Health State University (“OHSU”) and Peking (“PEKING”). We included ADHD-200 study-sites that contributed several functional runs per participant (NYU, OHSU) as well as a study-site with a single run (PEKING) whose total duration was at least 8 minutes. Raw, motion-uncorrected data in NIfTI format were downloaded. Participants with only 1 run at NYU or OHSU were not included in the current study. PEKING study-site consisted of Samples 1 and 2: from the National Key Laboratory of Cognitive Neuroscience and Learning, Beijing Normal University and Sample 3: from the Institute of Biophysics, Chinese Academy of Sciences (note that scan duration for all PEKING samples was identical: 8 min 6 s, 240 volumes over 1 run).

**Inclusion/exclusion criteria at the study-sites**

Study-site specific, detailed inclusion/exclusion criteria for each ADHD-200 site are reported at http://fcon_1000.projects.nitrc.org/indi/adhd200/). We note that criteria are similar, but not identical across all sites, and not directly comparable to those in the ABIDE database. All three sites included children participants with a diagnosis of Attention Deficit Hyperactivity Disorder (ADHD) and Typically Developing (TD) children. Inclusion in the ADHD diagnostic group common to all sites was establishment of ADHD diagnosis based on the administration of the Schedule of Affective Disorders and Schizophrenia for Children—Present and Lifetime Version (KSADS-PL)^29^ at NYU and PEKING and on KSADS (^35^; ^29^) at OHSU, administered to parents and children. Stimulant medication was withheld from ADHD participants for whom it was currently prescribed at all sites. Exclusion criteria for potential ADHD and TD participants across all three sites included contraindications to MRI scanning environment; site-specific criteria are detailed below. Additional information on the inclusion/exclusion criteria is listed at http://fcon_1000.projects.nitrc.org/indi/adhd200/.

*Exclusion criteria for ADHD and TD participants*

NYU excluded all potential participants who had “chronic medical conditions”. NYU excluded potential ADHD participants with “a conduct disorder, bipolar and major depressive disorders, and any psychotic disorders” ^36^. OSHU excluded potential participants with a “history of neurological illness, chronic medical problems, sensorimotor handicap, autistic disorder, mental retardation, or significant head trauma (with loss of consciousness) was identified by parent report, or if they had evidence of psychotic disorder or bipolar disorder on the structured parent psychiatric interview”. Typically developing (TD) control children were excluded for “presence of conduct disorder, major depressive disorder, or history of psychotic disorder, as well as for presence of ADHD” ^36^. OSHU also reported that participants “were excluded if they did not meet criteria for ADHD or non-ADHD groups (i.e. children deemed sub-threshold by the clinicians were excluded)” (http://fcon_1000.projects.nitrc.org/indi/adhd200/).

*Inclusion criteria for ADHD and TD participants*

NYU and PEKING required all children participants (ADHD and TD) to meet full-scale intelligence quotient (FSIQ) cut-off above 80. OHSU did not report IQ cutoff. Intelligence quotient (IQ) was estimated using the Wechsler Abbreviated Scale of Intelligence (WASI) (Wechler 1999) at NYU, using a three-subtest short form (Block Design, Vocabulary, and Information) of the Wechsler Intelligence Scale for Children, Fourth Edition (WISC-IV) (Wechler 2003) at OHSU, and using Wechsler Intelligence Scale for Chinese Children-Revised (WISCC-R) (Gong & Cai, 1993) at PEKING. NYU, OSHU and PEKING required all participants to be right-handed. PEKING required that all participants (ADHD and TD) had “no lifetime history of head trauma with loss of consciousness, (iii) no history of neurological disease and no diagnosis of either schizophrenia, affective disorder, pervasive development disorder, or substance abuse, on the basis of KSADS-PL administered to all participants”. For inclusion in the TD group, NYU required “absence of any Axis-I psychopathology on the basis parent and child KSADS-PL interview and T-scores <60 on Conners’ Parent Rating Scale-Revised, Long version (CPRS-R:LV)^37^ ADHD summary scales”. For all children, NYU required “absence of other chronic medical conditions” (http://fcon_1000.projects.nitrc.org/indi/adhd200/).

*Inclusion criteria for the ADHD group: establishment of ADHD diagnosis*

Inclusion of participants in the ADHD group required ascertainment of ADHD diagnosis, established on the basis of KSADS administration (“PL” version at NYU and PEKING and “I” version at OHSU) and the following criteria that varied by site. NYU established ADHD diagnosis by requiring potential participants to receive a diagnosis of ADHD on the basis of parent and child responses on the KSADS-PL and to obtain a T-score >=65 on “at least one ADHD related index” of the Conners’ Parent Rating Scale-Revised, Long version (CPRS-R: LV). NYU established ADHD subtypes “based on the interview and review of available records” ^36^. OHSU established ADHD diagnosis on the basis of KSADS-I, a parent and teacher Connors’ Rating Scale-3rd Edition ^38^, and via consensus diagnosis by a child psychiatrist and neuropsychologist. PEKING required potential participants (for inclusion in the ADHD group) to be first identified by achieving ADHD diagnosis via the Computerized Diagnostic Interview Schedule IV (C-DIS-IV) ^39^. PEKING then established final ADHD diagnosis by administering KSADS-PL. In addition, ADHD symptomology was assessed using the ADHD Rating Scale parent form (ADHD-RS) IV ^40^ (http://fcon_1000.projects.nitrc.org/indi/adhd200/).

**Datasets**

Datasets from 503 individuals were downloaded (N_ADHD_=232; N_TD_=271). The focus of the current analysis is on datasets from 443 participants for whom medication status was available (i.e., status noted as currently on psychotropic medication, or off psychotropic medication), N_ADHD_=175; N_TD_=268 (total N=443 datasets). None of the TD participants were currently taking psychotropic medication. Out of 175 ADHD participants, 114 were not currently on medication, while 61 were on some type of psychotropic medication. Specific medication class or name was not available in ADHD-200 database. The 443 datasets from the three sites break down as: NYU (N_ADHD_ =45; N_TD_ =86), OHSU (N_ADHD_ =28; N_TD_ =39), and PEKING (N_ADHD_ =102; N_TD_ =143) (Total: N_ADHD_ =175, N_TD_ =268).

**Demographic characteristics**

*ADHD datasets.* Participants at the three main sites (NYU, OHSU, PEKING) did not differ in age 11.40 (2.56) (mean and standard deviation; range: 7.17-17.96) for the ADHD group, and 11.60 (2.49) (range: 7.24-17.43) for the TD group (p=0.41). 172 ADHD participants were right-handed and 3 were left-handed; 260 TD participants were right-handed and 4 were left-handed. Scores were missing for 4 TD participants.

**Stimulant withholding information**

For participants in the ADHD group who were currently taking psychostimulant medications, NYU reported that such medications “were withheld at least 24 hours before scanning”. OHSU reported a “minimum washout of five half-lives” before children on short-acting stimulants were scanned (a period of 24-48 hours; ^36^). PEKING reported withholding of psychostimulants for “at least 48 hours prior to scanning” (http://fcon_1000.projects.nitrc.org/indi/adhd200/).

**Specific instructions to participants during the resting scan (eye status)**

OHSU: Participants were “instructed to stay still, and fixate on a standard fixation-cross in the center of the display” ^36^. NYU: Participants were “instructed to lie still and relax with their eyes open, while a standard fixation-cross was presented in the center of the display” ^36^. PEKING: Peking Sample 3: Participants were instructed to “simply remain still, close their eyes, think of nothing systematically, and not fall asleep” ^36^. Eye status information was not available for PEKING Samples 1 and 2 (http://fcon_1000.projects.nitrc.org/indi/adhd200/).

**MRI acquisition parameters**

BOLD signal was obtained with T2*-weighted echo planar imaging (EPI) sequence for all three ADHD-200 study-sites used in the current study. At NYU Child Study Center, EPI sequence parameters were: [TR (Repetition Time) /TE (Echo Time): 2000/15 ms, flip angle=90°, FOV (Field of View) =220 mm (read) x 192 mm (phase), 80 x 80 matrix, 33 contiguous axial 4 mm thick slices], with interleaved slice acquisition and no gap between slices. Scan duration of each run was 6 min (180 volumes), for a total scan duration at NYU of 12 min (360 volumes over 2 runs). At Oregon Health Sciences University (OHSU), EPI parameters were: [TR/TE: 2500/30 ms, flip angle=90°, FOV=240 mm (read) x 240 mm (phase), 64 x 64 matrix, 36 axial 3.8 mm slices], with interleaved slice acquisition and no gap between slices. Scan duration of each run was 3.32 min (82 volumes), for a total scan duration at OHSU of 9.96 min (246 volumes over 3 runs). PEKING was comprised of Samples 1 and 2, from the National Key Laboratory of Cognitive Neuroscience and Learning, Beijing Normal University and Sample 3, from the Institute of Biophysics, Chinese Academy of Sciences. At Peking Sample 1 (“PEKING_1”), EPI parameters were: [TR/TE: 2000/30 ms, flip angle=90°, FOV=200 mm (read) x 200 mm (phase), 64 x 64 matrix, 33 axial 3.5 mm slices], with interleaved slice acquisition and 0.7 mm gap between slices. At Peking Sample 2 (“PEKING_2”), EPI parameters were: [TR/TE: 2000/30 ms, flip angle=90°, FOV=200 mm (read) x 200 mm (phase), 64 x 64 matrix, 33 axial 3 mm slices], with interleaved slice acquisition and 0.6 mm gap between slices. At Peking Sample 3 (“PEKING_3”), EPI parameters were: [TR/TE: 2000/30 ms, flip angle=90°, FOV=200 mm (read) x 200 mm (phase), 64 x 64 matrix, 30 axial 4.5 mm slices], with interleaved slice acquisition and no gap between slices. Scan duration for all PEKING samples was 8 min 6 s (240 volumes over 1 run) (http://fcon_1000.projects.nitrc.org/indi/adhd200/).

**Head Movement Estimates**

The overall approach involves first linearizing the problem (expansions via smooth basis functions, polynomials and first order Taylor series) in order to find a least squares solution that minimizes the differences between the two images following simultaneous spatial and intensity transformations. We briefly outline key aspects of the approach; detailed treatment is given by Karl Friston and colleagues in^41^.

Here the problem is that of aligning a given volume image (“object” or “observed” image) so that it matches, as best as possible, another image (“template” or “reference” image). Potential differences between an object image and a reference image can be partitioned into two components: those due to (1) voxel intensity differences when the images are in full anatomical congruence with each other and (2) misalignment or spatial discrepancy^41^.

These differences are partitioned according to the expression, Equation 1 in Friston et al., 1995,


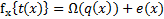
, (Equation 1)

where
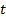
 is the template or reference image, and
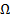
 is observed image;
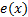
 is an error term. This expression says that the “two images can be approximated by applying an intensity transformation
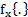
 to one and a spatial transformation
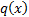
 to the other” (Friston et al., 1995). The
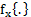
is an intensity operator that “maps voxel values from one image to another at point
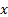
” while
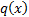
 denotes the spatial transformation between the two images^41^.

Low-order approximations and constraints are imposed on the form of
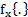
 and
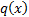
 simultaneously in order to linearize Equation 1 (so as to obtain a least squares solution)^41^. Specific constraints governing expansions “relate to preservation of local contiguity relationships and local stationariness of the intensity transformation”^41^. The two important constraints on
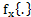
 and
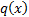
 are as follows:
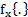
 can be “expressed in terms of a convolution and a (nonstationary) linear function and both
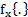
 and
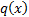
 change slowly with location”^41^. The characteristic that “
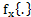
 can change with position but is similar within a given locale” ^42-44^ embodies the local stationariness assumption, meaning that “something does not change with position in the image” ^42-44^. Further, the fact that
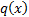
 undergoes slow changes means that the “spatial transformation is smooth and that local contiguity relationships are preserved” ^42-44^.

Equation #4 in ^41^ is read as follows, quoted from the paper: “One image can be approximated to another by (1) applying an intensity transformation to the first image (where the coefficients of the transformation expansion’s can change slowly with position), convolving, and (2) approximating the distortion of the second image by simply adding the effects of each component of the distortion (assuming the components are small relative to the image’s resolution”^41^. Because this equation is linear in the unknown coefficients (
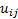
 and
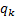
), these coefficients have a unique least squares solution^41^

Given
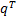
, 6 elements of the row vector
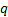
 “correspond to the estimated translations and rotations that constitute the movement to be corrected” ^41^(Friston et al., 1995). In SPM8, these are recorded as an “rp” file following rigid-body alignment step. Note that the linear (affine) position transformations specified for some portion of the image hold for all other portions of the image ^41^ by virtue of the treatment of this problem as one involving rigid body transformations.

# Supplementary Material References

1 Wu, D., Torres, E. B. & Jose, J. V. in *The Annual Meeting of the Society for Neuroscience.*

2 Nguyen, J., Majmudar, U., Papathomas, T. V., Silverstein, S. M. & Torres, E. B. Schizophrenia: The micro-movements perspective. *Neuropsychologia* **85**, 310-326, doi:10.1016/j.neuropsychologia.2016.03.003 (2016).

3 Torres, E. B. *et al.* Autism: the micro-movement perspective. *Front Integr Neurosci* **7**, 32, doi:10.3389/fnint.2013.00032 (2013).

4 Torres, E. B. & Lande, B. Objective and personalized longitudinal assessment of a pregnant patient with post severe brain trauma. *Front Hum Neurosci* **9**, 128, doi:10.3389/fnhum.2015.00128 (2015).

5 Torres, E. B. *et al.* Characterization of the Statistical Signatures of Micro-Movements Underlying Natural Gait Patterns in Children with Phelan McDermid Syndrome: Towards Precision-Phenotyping of Behavior in ASD. *Front Integr Neurosci* **10**, 22, doi:10.3389/fnint.2016.00022 (2016).

6 Torres, E. B. *et al.* Toward Precision Psychiatry: Statistical Platform for the Personalized Characterization of Natural Behaviors. *Front Neurol* **7**, 8, doi:10.3389/fneur.2016.00008 (2016).

7 Jerrell, J. M. Pharmacotherapy in the community-based treatment of children with bipolar I disorder. *Hum Psychopharmacol* **23**, 53-59, doi:10.1002/hup.900 (2008).

8 Loy, J. H., Merry, S. N., Hetrick, S. E. & Stasiak, K. Atypical antipsychotics for disruptive behaviour disorders in children and youths. *Cochrane Database Syst Rev* **9**, CD008559, doi:10.1002/14651858.CD008559.pub2 (2012).

9 Chang, K. D. The use of atypical antipsychotics in pediatric bipolar disorder. *J Clin Psychiatry* **69 Suppl 4**, 4-8 (2008).

10 Adler, B. A. *et al.* Drug-refractory aggression, self-injurious behavior, and severe tantrums in autism spectrum disorders: a chart review study. *Autism* **19**, 102-106, doi:10.1177/1362361314524641 (2015).

11 Ho, J. G. *et al.* The effects of aripiprazole on electrocardiography in children with pervasive developmental disorders. *J Child Adolesc Psychopharmacol* **22**, 277-283, doi:10.1089/cap.2011.0129 (2012).

12 McDougle, C. J., Stigler, K. A., Erickson, C. A. & Posey, D. J. Atypical antipsychotics in children and adolescents with autistic and other pervasive developmental disorders. *J Clin Psychiatry* **69 Suppl 4**, 15-20 (2008).

13 McCracken, J. T. *et al.* Risperidone in children with autism and serious behavioral problems. *N Engl J Med* **347**, 314-321, doi:10.1056/NEJMoa013171 (2002).

14 Schur, S. B. *et al.* Treatment recommendations for the use of antipsychotics for aggressive youth (TRAAY). Part I: a review. *J Am Acad Child Adolesc Psychiatry* **42**, 132-144 (2003).

15 Sikich, L., Hamer, R. M., Bashford, R. A., Sheitman, B. B. & Lieberman, J. A. A pilot study of risperidone, olanzapine, and haloperidol in psychotic youth: a double-blind, randomized, 8-week trial. *Neuropsychopharmacology* **29**, 133-145, doi:10.1038/sj.npp.1300327 (2004).

16 Lord, C. *et al.* The autism diagnostic observation schedule-generic: a standard measure of social and communication deficits associated with the spectrum of autism. *Journal of autism and developmental disorders* **30**, 205-223 (2000).

17 Lord, C., Rutter, M. & Le Couteur, A. Autism Diagnostic Interview-Revised: a revised version of a diagnostic interview for caregivers of individuals with possible pervasive developmental disorders. *Journal of autism and developmental disorders* **24**, 659-685 (1994).

18 Wechsler, D. (The Psychological Corporation, San Antonio, TX, 1997).

19 Wechsler, D. (The Psychological Corporation, San Antonio, Texas, 1999).

20 Wechsler, D. (The Psychological Corporation, San Antonio, TX, 2003).

21 Raven, J. C. (Oxford Psychological Press, Oxford, UK, 2000).

22 Dunn, L. M. & Dunn, L. M. (American Guidance Services, Circle Pines, MN, 1997).

23 Wechsler, D. (The Psychological Corporation, San Antonio, TX, 1991).

24 Spence, S. H. Structure of anxiety symptoms among children: A confirmatory factor-analytic study. *Journal of Abnormal Psychology* **106**, 280-297, doi:10.1037/0021-843X.106.2.280 (1997).

25 Achenbach, T. M. (University of Vermont Department of Psychiatry, Burlington, VT, 1997).

26 Constantino, J. N. & Gruber, C. P. (Western Psychological Services, Los Angeles, CA, 2005).

27 Sparrow, S. S., Cicchetti, D. V. & Balla, D. A. (American Guidance Service, Inc., Circle Pines, MN, 2005).

28 Conners, C. K., Erhart, D. & Sparrow, E. (Multi-Health Systems Inc., New York, NY, 1999).

29 Kaufman, J. *et al.* Schedule for Affective Disorders and Schizophrenia for School-Age Children-Present and Lifetime Version (K-SADS-PL): initial reliability and validity data. *Journal of the American Academy of Child and Adolescent Psychiatry* **36**, 980-988, doi:10.1097/00004583-199707000-00021 (1997).

30 First, M. B., Spitzer, R. L., Gibbon, M. & Williams, J. B. W. (Biometrics Research, New York State Psychiatric Institute, New York, 2002).

31 Adler, L. & Cohen, J. Diagnosis and evaluation of adults with attention-deficit/hyperactivity disorder. *The Psychiatric clinics of North America* **27**, 187-201, doi:10.1016/j.psc.2003.12.003 (2004).

32 Rutter, M., Bailey, A. & Lord, C. (Western Psychological Services, Los Angeles, 2003).

33 Oldfield, R. C. The assessment and analysis of handedness: the Edinburgh inventory. *Neuropsychologia* **9**, 97-113 (1971).

34 Annett, M. A classification of hand preference by association analysis. *British Journal of Psychology* **61**, 303-321, doi:10.1111/j.2044-8295.1970.tb01248.x (1970).

35 Chambers, W. J. *et al.* The assessment of affective disorders in children and adolescents by semistructured interview. Test-retest reliability of the schedule for affective disorders and schizophrenia for school-age children, present episode version. *Arch Gen Psychiatry* **42**, 696-702 (1985).

36 Fair, D. A. *et al.* Distinct neural signatures detected for ADHD subtypes after controlling for micro-movements in resting state functional connectivity MRI data. *Front Syst Neurosci* **6**, 80, doi:10.3389/fnsys.2012.00080 (2012).

37 Farre-Riba, A. & Narbona, J. [Conners' rating scales in the assessment of attention deficit disorder with hyperactivity (ADHD). A new validation and factor analysis in Spanish children]. *Rev Neurol* **25**, 200-204 (1997).

38 Conners, C. K. *Conners 3rd Edition Manual*. (Multi-Health Systems Inc, 2008).

39 Robins, L. N., Helzer, J. E., Croughan, J. & Ratcliff, K. S. National Institute of Mental Health Diagnostic Interview Schedule. Its history, characteristics, and validity. *Arch Gen Psychiatry* **38**, 381-389 (1981).

40 DuPaul, G. J., Power, T. J., Anastopoulos, A. D. & Reid, R. *ADHD rating scale 5 for children and adolescents : checklists, norms, and clinical interpretation*. (The Guilford Press, 2016).

41 Friston, K. J. *et al.* Spatial Registration and Normalization of Images. *Human Brain Mapping* **2**, 165-189 (1995).

42 Friston, K. J., Frith, C. D., Frackowiak, R. S. & Turner, R. Characterizing dynamic brain responses with fMRI: a multivariate approach. *Neuroimage* **2**, 166-172 (1995).

43 Friston, K. J., Frith, C. D., Turner, R. & Frackowiak, R. S. Characterizing evoked hemodynamics with fMRI. *Neuroimage* **2**, 157-165 (1995).

44 Friston, K. J. *et al.* Analysis of fMRI time-series revisited. *Neuroimage* **2**, 45-53, doi:10.1006/nimg.1995.1007 (1995).
